# Heterozygous variants in SIX3 and POU1F1 cause pituitary hormone deficiency in mouse and man

**DOI:** 10.1093/hmg/ddac192

**Published:** 2022-08-11

**Authors:** Hironori Bando, Michelle L Brinkmeier, Frederic Castinetti, Qing Fang, Mi-Sun Lee, Alexandru Saveanu, Frédérique Albarel, Clémentine Dupuis, Thierry Brue, Sally A Camper

**Affiliations:** Department of Human Genetics, University of Michigan, Ann Arbor, MI, USA; Department of Human Genetics, University of Michigan, Ann Arbor, MI, USA; Assistance Publique-Hôpitaux de Marseille (AP-HM), Department of Endocrinology, Hôpital de la Conception, Centre de Référence des Maladies Rares de l’hypophyse HYPO, Marseille, France; Aix-Marseille Université, Institut National de la Santé et de la Recherche Médicale (INSERM), U1251, Marseille Medical Genetics (MMG), Institut Marseille, Maladies Rares (MarMaRa), Marseille, France; Department of Human Genetics, University of Michigan, Ann Arbor, MI, USA; Michigan Neuroscience Institute, Department of Biological Chemistry, University of Michigan, Ann Arbor, MI, USA; Assistance Publique-Hôpitaux de Marseille (AP-HM), Department of Endocrinology, Hôpital de la Conception, Centre de Référence des Maladies Rares de l’hypophyse HYPO, Marseille, France; Aix-Marseille Université, Institut National de la Santé et de la Recherche Médicale (INSERM), U1251, Marseille Medical Genetics (MMG), Institut Marseille, Maladies Rares (MarMaRa), Marseille, France; Assistance Publique-Hôpitaux de Marseille (AP-HM), Department of Endocrinology, Hôpital de la Conception, Centre de Référence des Maladies Rares de l’hypophyse HYPO, Marseille, France; Aix-Marseille Université, Institut National de la Santé et de la Recherche Médicale (INSERM), U1251, Marseille Medical Genetics (MMG), Institut Marseille, Maladies Rares (MarMaRa), Marseille, France; Department of Pediatrics, Centre Hospitalier Universitaire de Grenoble-Alpes, site Nord, Hôpital Couple Enfants, Grenoble, France; Assistance Publique-Hôpitaux de Marseille (AP-HM), Department of Endocrinology, Hôpital de la Conception, Centre de Référence des Maladies Rares de l’hypophyse HYPO, Marseille, France; Aix-Marseille Université, Institut National de la Santé et de la Recherche Médicale (INSERM), U1251, Marseille Medical Genetics (MMG), Institut Marseille, Maladies Rares (MarMaRa), Marseille, France; Department of Human Genetics, University of Michigan, Ann Arbor, MI, USA

## Abstract

Congenital hypopituitarism is a genetically heterogeneous condition that is part of a spectrum disorder that can include holoprosencephaly. Heterozygous mutations in *SIX3* cause variable holoprosencephaly in humans and mice. We identified two children with neonatal hypopituitarism and thin pituitary stalk who were doubly heterozygous for rare, likely deleterious variants in the transcription factors SIX3 and POU1F1. We used genetically engineered mice to understand the disease pathophysiology. *Pou1f1* loss-of-function heterozygotes are unaffected; *Six3* heterozygotes have pituitary gland dysmorphology and incompletely ossified palate; and the *Six3*^+/−^; *Pou1f1*^+/dw^ double heterozygote mice have a pronounced phenotype, including pituitary growth through the palate. The interaction of *Pou1f1* and *Six3* in mice supports the possibility of digenic pituitary disease in children. Disruption of *Six3* expression in the oral ectoderm completely ablated anterior pituitary development, and deletion *of Six3* in the neural ectoderm blocked the development of the pituitary stalk and both anterior and posterior pituitary lobes. *Six3* is required in both oral and neural ectodermal tissues for the activation of signaling pathways and transcription factors necessary for pituitary cell fate. These studies clarify the mechanism of SIX3 action in pituitary development and provide support for a digenic basis for hypopituitarism.

## Graphical Abstract

**Graphical Abstract:**
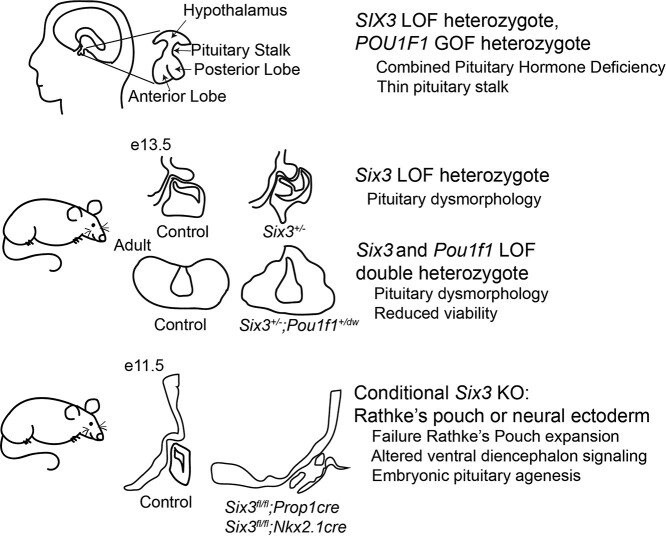

## Introduction

The anterior and posterior lobes of the pituitary gland develop from oral and neural ectoderm, respectively, under the control of embryonic signaling pathways and lineage-specific transcription factors. Variants in more than 60 genes have been reported in patients with combined pituitary hormone deficiency (CPHD), and most of the genes have roles in pituitary and/or hypothalamic development (1–23). Because variants in known genes only explain ~16% of CPHD cases, additional genes are likely important for pituitary development and disease (24).

Pituitary stalk interruption syndrome (PSIS) is a congenital midline defect defined as thinning or disappearance of the pituitary stalk, hypoplasia of the anterior pituitary and ectopic posterior pituitary (25). PSIS is associated with growth hormone deficiency (GHD) that usually progresses to CPHD (26–30). Approximately 7.8% of individuals with GHD have PSIS (31). A thin pituitary stalk may be a mild form of PSIS. Pituitary injury and stalk rupture during delivery had been thought to cause PSIS (32). However, several genes are now implicated in PSIS, including orthodenticle homeobox 2 (*OTX2*), aryl hydrocarbon receptor nuclear translocator 2 (*ARNT2*), chromodomain helicase DNA binding protein 7 (*CHD7*), paired box 6 (*PAX6*), roundabout guidance receptor 1 (*ROBO1*) and LIM homeobox 4 (*LHX4*) (33). The extent of genetic heterogeneity underlying PSIS is not known.

Holoprosencephaly (HPE) is a developmental defect characterized by a failure of the forebrain to cleave into cerebral hemispheres and ventricles during the prenatal period (34). This defect has a range of phenotypes from severe to mild: alobar, semi-lobar, lobar and middle interhemispheric variant (35). Pituitary hormone deficiencies are present in 63% of non-chromosomal, non-syndromic HPE patients (36). Mutations in at least 17 genes have been reported in the patients of HPE (37). Several of them are involved in sonic hedgehog (SHH) signaling, including *SHH,* Sine oculis homeobox homolog 3 *(SIX3),* zinc finger protein of the cerebellum 2 *(ZIC2)*, TGFB induced factor homeobox 1 *(TGIF1)* and patched 1 *(PTCH1)* (38). Mutations in some HPE-related genes (*SHH* and *TGIF*) also cause PSIS (39).

SIX3 variants cause HPE with incomplete penetrance and variable phenotypic presentation in mouse and man (40–42). Burden tests indicate that oligogenic inheritance is a significant contributor to HPE, and this likely impacts the expressivity (37). In addition, environmental factors, including maternal alcohol consumption or dietary insufficiencies, can interact with genetic risk factors to enhance disease susceptibility (43). A genetic interaction of *Six3* and *Hesx1,* a transcription factor associated with CPHD and septo-optic dysplasia, has been reported in mice (44). These doubly heterozygous mice exhibit growth insufficiency, hypopituitarism and pituitary dysmorphology not observed in single heterozygotes, consistent with digenic interactions. Although doubly heterozygous patients have not yet been reported with *SIX3* and *HESX1* variants (45), heterozygous deletion of *SIX3* has been observed in HPE patients with variants in genes related to the primary cilium and SHH signaling (37).

SIX3 is crucial for the formation of the forebrain and eye through its roles as both a transcriptional activator and repressor (46). In the forebrain, SIX3 functions as a repressor of *Wnt1* gene expression, preventing differentiation into more posterior cell fates, and SIX3 activates expression of sonic hedgehog (SHH) and *Foxg1* (42). In the eye, SIX3 represses *Wnt8b* and activates *Pax6,* a key driver of eye development, as well as SHH. Studies in mice and cell lines have demonstrated that SIX3 is important for development of hypothalamic neurons, fertility and growth (47,48), but more studies are necessary to understand the mechanism of SIX3 action and role in pituitary disease.

We found doubly heterozygous variants in SIX3 and POU domain class 1 transcription factor 1 (POU1F1, also known as PIT-1) in two children with PSIS and CPHD. Here we report that *Six3* and *Pou1f1* can interact in mice to intensify the severity of pituitary defects, and we demonstrate that SIX3 expression in both the neural ectoderm and oral ectoderm is important for pituitary gland development. Thus, more cases of CPHD may be caused by digenic or oligogenic disease than previously appreciated.

## Results

### CPHD patients have a *SIX3* variant with incomplete penetrance

**Figure 1:**
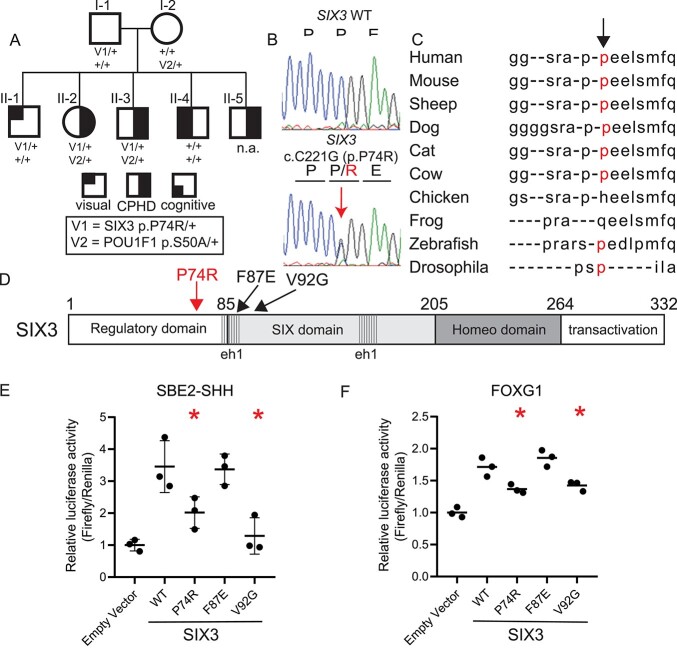
Children with CPHD have a *SIX3* variant that reduces transactivation. (**A**) Pedigree of three children with CPHD. Heterozygotes are indicated for variants in SIX3 (p.P74R) and POU1F1 (p.S50A) inherited from their unaffected father and mother, respectively. (**B**) Sanger sequence validated the heterozygous variant in *SIX3* (c.C221G) and segregation within the pedigree, including an unaffected sibling heterozygous for the *SIX3* variant. (**C**) The proline at codon 74 of SIX3 is conserved in mammals. (**D**) SIX3 p.P74R is in the regulatory domain N-terminal to other variants associated with HPE. SIX3 p.F87E affects repressor activity and V92G affects transactivation. (**E**) Cells were transfected with SIX3 expression vectors bearing the indicated amino acid substitutions and a reporter gene, *SBE2-Luc*. (**F**) Same as E except the expression vector was *Foxg1-Luc*. Asterisks indicate a significant difference from wild type (WT) (*P* < 0.05).

We conducted exome sequencing analysis on a French familial case of thin pituitary stalk and CPHD (Fig. 1A). Three children were affected with hypopituitarism and presented with neonatal growth hormone (GH) and thyroid stimulating hormone (TSH) deficiency, and they responded well to hormone-replacement therapy (Table 1). The two older affected children were doubly heterozygous for rare, likely deleterious variants: *SIX3* p.P74R/+ and *POU1F1* p.S50A/+ (Fig. 1B). No DNA was available from the younger child with CPHD. These *SIX3* and *POU1F1* variants were inherited from the unaffected father and mother, respectively. There were two children with no obvious pituitary hormone deficiency. One carried the *SIX3* variant and had congenital nystagmus and severe visual impairment. The other one had congenital nystagmus, retinal dystrophy, cognitive impairment and autism.

**Table 1:** Clinical assessment of children reveals GH and thyroid hormone deficiencies

Individual	II-2	II-3	II-5
Weeks gestation at birth	41 + 6 days	40	38 + 2 days
Birth length (cm)^a^	45 (−2.9)	46 (−2.4)	47 (−1.5)
Birth weight (kg)^a^	3.15 (−0.4)	3.42 (−0.1)	2.81 (−1.0)
Age at diagnosis	Day 1	Day 1	Day 1
Symptoms at diagnosis	Hypoglycemia	Hypoglycemia	Hypoglycemia
*Basal hormone levels*			
TSH (microUI/l)	0.03	0.02	n.a.
T4/L (pmol/l)	1.5	2.2	3.7
T3/L (pmol/l)	1.5	1.7	1.6
IGF1 (ng/l)	<5	10	5
*Hypoglycemic hormone levels*			
GH (ng/ml)	<0.03	0.03	n.a.
ACTH (pmol/l)	4.1	n.a.	n.a.
Cortisol 9 nmol/l)	727	701	1098
MRI			
Anterior pituitary	Small, 3 mm	Normal, 6 mm	n.a.
Pituitary stalk	Thin, continuous	Thin, continuous	n.a.
Olfactory bulb	Small	Normal	n.a.

n.a. = not available.

^a^The results are given with standard deviation from normal in parenthesis.

The *POU1F1* p.S50A/+ and *SIX3* p.P74R/+ variants affect function in cell culture assays. The *POU1F1* p.S50A/+ variant affects splicing, resulting in production of the repressive POU1F1 beta isoform, instead of the activating isoform, POU1F1 alpha (49). Variants that cause a shift in POU1F1 isoforms result in incompletely penetrant CPHD or isolated growth hormone deficiency (IGHD) without other significant phenotypes (49–51). The SIX3 p.P74R variant is present at a very low allele frequency, 0.0185% (46/248636 heterozygotes and no homozygotes in gnomad) (52). The proline is conserved among mammals (Fig. 1C). Mutation Taster predicted SIX3 p.P74R as ‘disease causing’ (53), and the combined annotation dependent depletion (CADD) score of this variant is 19.51 [32]. The *SIX3* p.P74R variant (Fig. 1D) was identified in five individuals with intellectual disability and various other features in four unrelated families, but it was classified as benign because it was also present in unaffected family members (unpublished data, variants and data shared through Mobidetails) (54).

We assessed the functional significance of the SIX3 variant on two of its well-established down-stream target genes using cell transfection assays. SIX3 activates the *SHH* brain enhancer-2 (SBE2), and mutations in either the *SHH* enhancer or SIX3 can cause HPE (55). SIX3, together with SHH, activates early expression of the forkhead box g1 gene (*FOXG1*), and this process is evolutionarily conserved (56,57). SIX3 p.P74R has significantly weaker activation activity than wild type on both the SHH and FOXG1 luciferase reporter genes. The SIX3 p.P74R variant has the same activity as the p.V92G variant, which was reported in alobar HPE (58) (Fig. 1E and F). SIX3 represses wingless related integration site gene family (WNT) signaling, and a previously reported loss of function mutation in the interaction domain for the co-repressor TLE (SIX3 p.F87E) reduces repressor activity (55). The p.F87E variant retained WT transactivation of SBE2, as expected. Based on these assays, the p.P74R variant appears to weaken the activity of SIX3-mediated transcriptional activation.

### SIX3 is expressed in progenitors in the developing pituitary gland and hypothalamus

SIX3 is expressed in the ventral diencephalon, which forms the hypothalamus, the evaginating neural ectoderm forming the infundibulum and pituitary stalk, and the invaginating oral ectoderm forming Rathke’s pouch, at E10.5 (Supplementary Material, Fig. S1A). This expression pattern is consistent throughout early developmental stages, E11.5-E13.5, with the highest SIX3 expression in ventral diencephalon, the infundibulum and in the dorsal aspect of Rathke’s pouch. SIX3 expression is reduced after E13.5, and SIX3 immunostaining is detected in only a few cells in the anterior lobe of the pituitary gland at P7. The spatial and temporal expression of *Six3* suggests that it could be involved in the development of the hypothalamus and/or the pituitary gland, including the pituitary stalk and both the anterior and posterior lobes.

**Figure 2:**
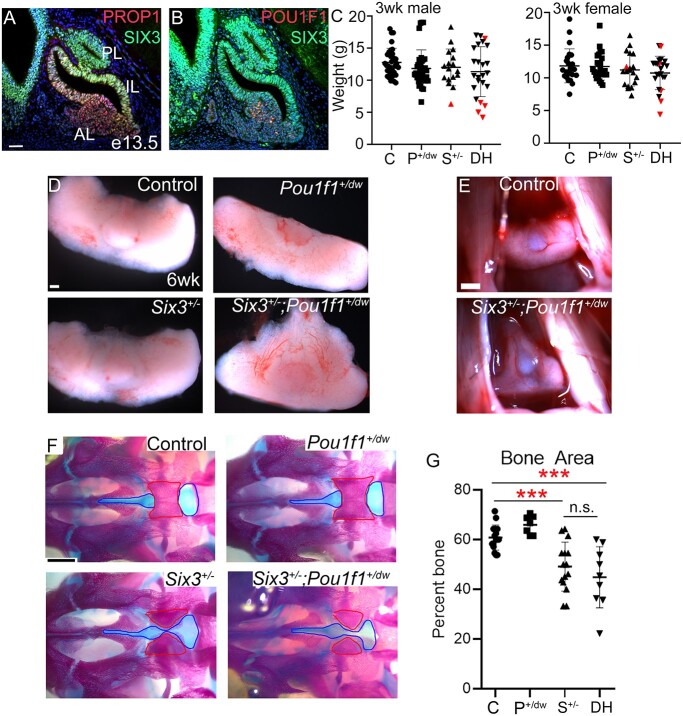
*Six3^+/−^*; *Pou1f1*^+/dw^ double heterozygous mice exhibit pituitary dysmorphology and impaired palate ossification. (**A**) Immunostaining reveals SIX3 colocalization with PROP1 at e13.5. (**B**) SIX3 and POU1F1 only co-localize in a few cells. (**C**) *Six3*^+/−^; *Pou1f1^+/dw^* mice weigh the same at 3 weeks compared with single mutants and controls. Each symbol represents an individual mouse. Symbols colored in red represent mice that did not survive past weaning. (**D**) Whole pituitary glands dissected at 6 weeks show abnormal shape of the *Six3*^+/−^; *Pou1f1^+/dw^* double heterozygotes. (**E**) Images of the pituitary gland within the head with rostral aspects at the top. (**F**) Ventral view of skeletal preps from P0 pups. Red outline indicates area of presphenoid bone that is ossified, and blue outline indicates area of palatal cartilage measured. (**G**) Bone area was calculated for each individual and genotype as a percentage of bone (red outline) compared with the overall area of bone plus cartilage (blue outline). Each symbol represents an individual neonate. Statistical significance was determined using a Student *t* test. *** = *P* < 0.001. C = control, P*^+/dw^* = *Pou1f1^+/dw^*, S*^+/−^* = *Six3*^+/−^, DH = *Six3*^+/−^; *Pou1f1^+/dw^*. Scale bar in panel A represents 50 μm and is applicable to panels A and B. Scale bar in panel D represents 100 μm. The scale bar in panels E and F represents 500 μm.

We analyzed the co-localization of SIX3 with other critical pituitary transcription factors. SIX3 and the pituitary progenitor marker, PROP1, exhibit substantial overlap at E13.5 (Fig. 2A). The cells co-expressing SIX3 and PROP1 are in the region of pituitary primordium where stem cells undergo an epithelial to mesenchymal-like transition and initiate differentiation (59). PROP1 is necessary for this process as well as for activation of POU1F1, the signature transcription factor essential for the lineage composed of growth hormone, thyrotropin and prolactin producing cells (60). POU1F1 co-localizes with only a few SIX3 expressing cells at E13.5, which is the onset of POU1F1 expression (Fig. 2B) (44). The co-localization of SIX3 with PROP1 suggests that SIX3 could influence development and/or function of GH and TSH cells by affecting progenitor differentiation into POU1F1 expressing cells.

### *Six3*; *Pou1f1* double heterozygotes have abnormal pituitary and palate morphology

POU1F1 is a pituitary-specific transcription factor, and mutations in POU1F1 cause non-syndromic CPHD or IGHD (61). Dominant and recessive variants have been reported. To assess the potential for digenic interaction of SIX3 and POU1F1 leading to hypopituitarism, we analyzed *Six3^+/−^*; *Pou1f1*^+/dw^ mice. The *Pou1f1*^dw^ allele is a p.W251C missense variant in the homeodomain that abrogates DNA binding and results in recessive pituitary insufficiency in *Pou1f1*^dw/dw^ mice; heterozygotes are unaffected (62). Mice doubly heterozygous for the *Six3* and *Pou1f1* mutant alleles are observed in expected Mendelian proportions at weaning (*N* = 359, *P* = 0.259). There is no significant difference in the weight of male or female *Six3^+/−^; Pou1f1*^+/dw^ double heterozygotes compared with single heterozygotes and wild-type controls at 3 or 6 weeks (*P* = 0.11–0.87), (Fig. 2C and Table 2). However, the double heterozygotes had significantly reduced viability (12% moribund) compared with *Six3^+/−^* (1%) or the other genotypes (0% wild type, 0% *Pou1f1*^dw/+^) (*P* = 0.0094). The animals with poor viability appeared sickly and frail and/or had hydrocephaly. We analyzed pituitaries of surviving mice at 6 weeks (*N* = 106). Modest abnormalities were noted in some *Six3^+/−^* mice (12%). However, more obvious pituitary dysmorphology, including extension of pituitary tissue through the palate, was observed in 34% of *Six3^+/−^*; *Pou1f1*^+/dw^ mutants (Fig. 2D and E, Table 2).

**Table 2:** Phenotypes of *Pou1f1*^+/dw^ and *Six3^+/−^* single and double heterozygotes

**Phenotype**	**Age** (weeks)	**Genotype observed/total** (%)				***P*-value** ^a^
		**wild type**	***Pou1f1*** ^***+/dw***^	***Six3*** ^***+/−***^	***Pou1f1*** ^***+/dw***^**; *Six3*** ^***+/−***^	
Moribund	3	0/106 (0)	0/86 (0)	1/82 (1)	10/85 (12)	0.0001, s.
Hydrocephaly	3	0/106 (0)	0/86 (0)	7/82 (9)	5/85 (6)	0.0017, s.
Pituitary dysmorphology	6	0/24 (0)	0/24 (0)	2/17 (12)	14/41 (34)	0.0001, s.
Weight (g ± std. error) male female	6	28.0 ± 3.0	25.7 ± 2.5	25.8 ± 1.1	26.7 ± 3.7	0.2265, n.s.
		20.6 ± 2.5	20.9 ± 3.1	20.5 ± 3.6	18.5 ± 2.0	0.313, n.s.

^a^n.s. = not significant, s. = significant.

Palate closure should be complete by birth (63,64). We used alizarin red and alcian blue staining to assess the degree of palate ossification in newborns (*N* = 48) (Fig. 2F). The palate was less ossified in both *Six3^+/−^* single mutants and *Six3^+/−^*; *Pou1f1*^+/dw^ double heterozygotes relative to other genotypes. The ossified area of the pre-sphenoid bone was reduced and the cartilage area was increased in both *Six3^+/−^* (*N* = 7, 49 ± 10%, *P* = 0.0005) and *Six3^+/−^*; *Pou1f1*^+/dw^ mice (N = 10, 45 ± 12%, *P* = 0.002) compared with either wild type (*N* = 16, 61 ± 5%) or *Pou1f1*^+/dw^ mice (*N* = 7, 66 ± 4%) (Fig. 2G). Thus, SIX3 deficiency delays palatal ossification.

**Figure 3:**
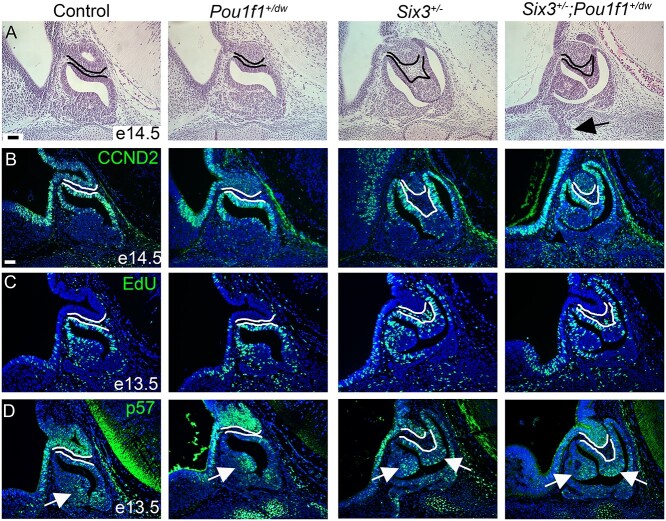
*Six3* haploinsufficiency causes dysmorphology. (**A**) Representative hematoxylin and eosin staining is shown for sagittal sections from e14.5 embryos of each genotype. The posterior lobe and dorsal aspect of Rathke’s pouch are outlined. Cells between these tissues are present in *Six3*^+/−^ and *Six3*^+/−^; *Pou1f1^+/dw^* embryos. Arrow shows growth of Rathke’s pouch through the underlying cartilage plate. (**B**) Representative immunostaining for CCND2 is shown for each genotype, *Six3*^+/−^ (*N* = 3) and *Six3*^+/−^; *Pou1f1^+/dw^* (*N* = 5). (**C**) Staining for EdU is shown for embryos collected at e13.5, *Six3*^+/−^ (*N* = 6) and *Six3*^+/−^; *Pou1f1^+/dw^* (*N* = 5). (**D**) Immunostaining for p57 reveals cells exiting the cell cycle, arrows; *Six3^+/−^* (*N* = 6) and *Six3^+/−^*; *Pou1f1^+/dw^* (*N* = 5). The scale bar in panels A and B represents 50 μm and is applicable to all panels.

To trace the origin of the pituitary dysmorphology, we analyzed sections from E14.5, postnatal day 0 (P0) and adult mouse pituitaries. The neural and oral ectoderm are normally juxtaposed throughout pituitary development, but both *Six3^+/−^* and *Six3^+/−^*; *Pou1f1*^+/dw^ embryos had an abnormal number of mesenchymal cells between these tissue layers and a highly bifurcated marginal zone at E14.5, consistent with an epithelial to mesenchymal transition defect (Fig. 3A). At E14.5, the marginal zone contains SOX2 expressing stem cells that are proliferating (65). Cells in this zone express CCND2 and PITX2, indicating active proliferation and commitment to pituitary fate, respectively (Fig. 3B and Supplementary Material, Fig. S1B). We analyzed proliferation by 5-ethynyl-2′-deoxyuridine (EdU) labeling. We observed a lack of EdU labeled cells dorsal to the lumen in the bifurcated marginal zones of both *Six3^+/−^* and *Six3^+/−^*; *Pou1f1*^+/dw^ embryos (Fig. 3C). Considering the dysmorphology in *Six3^+/−^* and *Six3^+/−^*; *Pou1f1*^+/dw^ embryos, the overall pattern of CCND2 and EdU immunostaining suggests that dorsal-ventral patterning is generally maintained, with the cells farthest from the signaling center ceasing proliferation. However, cells expressing *Cdkn1c* (p57), a marker of cell cycle exit, are normally concentrated in a small area on the ventral side of the lumen, and both *Six3^+/−^* and *Six3^+/−^*; *Pou1f1*^+/dw^ mutants have abundant p57 positive cells on the dorsal side of the lumen (Fig. 3D). We did not observe any dramatic changes in *Axin2*, *Ctgf or Cyr61* expression by in situ hybridization at E14.5, suggesting that WNT and Hippo signaling are intact at that time (Supplementary Material, Fig. S1C–E). There was no significant difference in proliferation amongst the genotypes at E13.5 (Supplementary Material, Table S1).

Each of the expected hormone-producing cell types was present in both newborn (Supplementary Material, Fig. S2) and adult (Supplementary Material, Fig. S3) mouse pituitaries, including the anterior lobe cells that produce GH, adrenocorticotropin (ACTH), luteinizing hormone (LH) and TSH and the arginine vasopressin (AVP) neurons that project to the posterior lobe. The cells present in the abnormal area of the organ were mostly hormone negative, although a few scattered GH and ACTH positive cells were detected. Thus, *Six3* haploinsufficiency can substantially affect pituitary morphology and growth, and haploinsufficiency for both *Six3* and *Pou1f1* exacerbates the phenotype.

### Rathke’s pouch specific *Six3* knockout causes pituitary aplasia

To investigate the mechanism of *Six3* function in pituitary development, we used a well-characterized *Prop1-cre* bacterial artificial chromosome transgenic mouse line to disrupt *Six3* in Rathke’s pouch (66). We confirmed that cre excision was specific to Rathke’s pouch using lineage tracing and immunostaining for SIX3 (Supplementary Material, Fig. S4A). Embryos carrying *Prop1-cre* and the *Rosa tdTomato* reporter gene (B6.Cg-*Gt(ROSA)26Sortm9(CAG-tdTomato)Hze*/J) exhibited tdTomato expression in Rathke’s pouch and not the adjacent ventral diencephalon. In addition, SIX3 immunostaining was not detected in Rathke’s pouch of *Six3^fl/fl^; Prop1-cre* mutants at E12.5, but it was present the ventral diencephalon (*N* = 7) and E11.5 (*N* = 14) (Supplementary Material, Fig. S4B and C).

**Figure 4:**
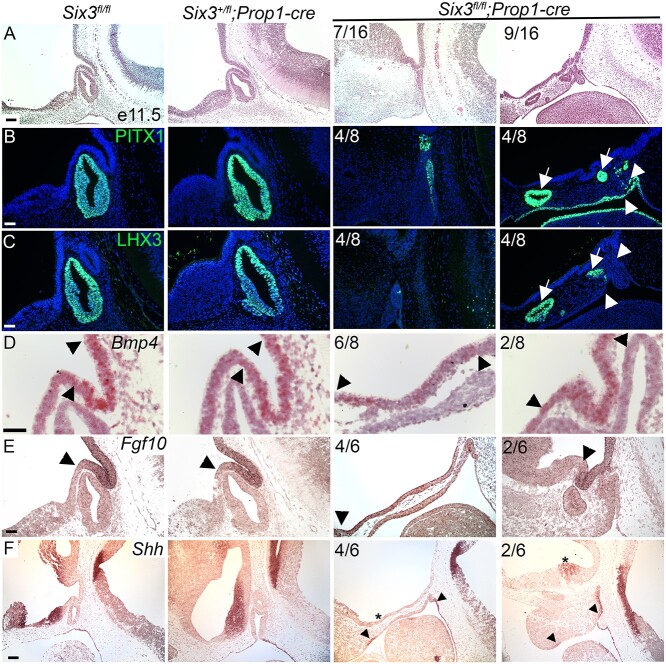
Pituitary-specific deletion of *Six3* results in Rathke’s pouch hypoplasia and impaired ventral diencephalon signaling. (**A**) Sagittal sections of embryos representing three genotypes were collected at e11.5 and stained with hematoxylin and eosin. The *Six3^fl/fl^*; *Prop1-cre* embryos had a range of abnormalities from hypoplastic Rathke’s pouch with no evidence of infundibulum formation (7/16) to multiple invaginations at ectopic sites (9/16). (**B**) Sections from the same genotypes and age were immunostained for PITX1 and (**C**) LHX3. Some regions of *Six3^fl/fl^*; *Prop1-cre* embryos expressed both PITX1 and LHX3 (arrows), and other regions expressed PITX1, but without detectable LHX3 immunostaining (arrowheads). (**D**) Sections from the same genotypes and age were used for *in situ* hybridization for *Bmp4* with RNAscope probes*,* and traditional in situ probes for (**E**) *Fgf10* and (**F**) *Shh.* Boundaries of *Bmp4* and *Fgf10* expression are expanded in *Six3^fl/fl^*; *Prop1-cre* embryos relative to controls (arrowheads). *Shh* transcripts represent in both the ventral diencephalon and oral ectoderm in controls, but *Six3^fl/fl^*; *Prop1-cre* mutants had reduced or absent *Shh* staining in the ventral diencephalon (asterisks) despite detectable staining in the oral ectoderm (arrowheads). Two examples from the *Six3^fl/fl^*; *Prop1-cre* mutants are shown for each marker, with the left panel representing the more severe phenotype. The fraction of embryos reflecting the severe and milder phenotypes is indicated. The scale bar in panel A represents 100 μm. The scale bar in panel B–F represents 50 μm.

Rathke’s pouch was thin at E10.5 in *Six3^fl/fl^; Prop1-cre* mutants, and LHX3 expression was absent (*N* = 4/5) or markedly reduced (*N* = 1/5), indicating early disruption of pituitary fate specification (Supplementary Material, Fig. S4D). At E11.5 the phenotype was variable ranging from a thin, hypoplastic pouch (*N* = 7/16) to multiple ectopic invaginations extending rostrally along the oral ectoderm (*N* = 9/16) (Fig. 4A). To determine whether cells in these areas were committed to pituitary fate, we assessed expression of early-acting pituitary transcription factors PITX1 and LHX3 by immunostaining. We detected PITX1 staining in all *Six3^fl/fl^; Prop1-cre* mutants at E11.5, and LHX3 immunostaining in some mutants (*N* = 4/8) (Fig. 4B and C). The more severely affected mutants had Rathke’s pouch hypoplasia, fewer PITX1 positive cells and only a few cells expressing LHX3. Thus, disruption of *Six3* in Rathke’s pouch reduces expression of both PITX1 and LHX3, with a stronger effect on LHX3.

*Six3* can interact genetically with *Hesx1,* causing activation of WNT signaling and hypopituitarism associated with pituitary gland dysmorphology (44). Because both SIX3 and HESX1 bind TLE family corepressors, we analyzed expression of *Hesx1* and TLE3 (Supplementary Material, Fig. S4E and F). *Hesx1* transcripts were detected throughout Rathke’s pouch in both control and *Six3^fl/fl^; Prop1-cre* mice, consistent with the expectation that *Hesx1* expression is independent of SIX3 (44). TLE3 is normally expressed in aspects of the ventral diencephalon and throughout Rathke’s pouch. Taking into account the morphological abnormalities in the mutants, TLE3 expression appeared normally patterned in control and *Six3^fl/fl^; Prop1-cre* mice at E11.5.

The co-repressor TLE4 is expressed in the developing infundibulum, and it interacts with SIX3 and other transcription factors to mediate repression (67). TLE4 expression and the evagination of the ventral diencephalon are normal in some *Six3^fl/fl^*; *Prop1-cre* mice (*N* = 4/7) at E12.5 (Supplementary Material, Fig. S5A). The more severely affected mutants have no evidence of evagination, and TLE4 expression is expanded rostrally along the ventral diencephalon. This suggests that the expression of SIX3 in the dorsal aspect of Rathke’s pouch is important for signaling evagination of the neural ectoderm. The nature of such signaling is not known (68).

Bone morphogenetic protein (BMP), fibroblast growth factor (FGF), SHH and WNT signaling are important for induction of Rathke’s pouch (69). We analyzed expression of *Bmp4*, *Fgf10*, *Shh* and *Axin2*. Both *Bmp4* and *Fgf10* transcripts are properly restricted to the infundibulum at E11.5 in controls and *Six3^+/fl^*; *Prop1-cre* mice (Fig. 4D and E). The pattern of *Bmp4* and *Fgf10* expression is normal in the *Six3^fl/fl^; Prop1-cre* mutants that have a defined infundibulum, but the expression domain is expanded in mutants that lack evagination. SHH is normally expressed in the ventral diencephalon, rostral to the domains that express BMP and FGF (Fig. 4F). *Shh* transcripts were either absent (*N* = 4/6) or reduced (*N* = 2/6) in *Six3^fl/fl^; Prop1-cre* mutants. The canonical WNT signaling pathway stabilizes β-catenin and activates downstream genes such as *Axin2*. *Axin2* is expressed in the dorsal hypothalamus, and there were no differences in expression among genotypes (Supplementary Material, Fig. S5B). *Six6* is normally expressed in the ventral diencephalon and Rathke’s pouch. There were also no differences in *Six6* expression among genotypes, indicating no compensatory upregulation of expression in mutants (Supplementary Material, Fig. S5C). Thus, SIX3 deficiency in Rathke’s pouch reduces *Shh* expression in the ventral diencephalon.

We analyzed the progression of Rathke’s pouch expansion at later gestational stages. At E12.5, *Six3^fl/fl^; Prop1-cre* mutants continue to exhibit varying levels of rudimentary pouch development with multiple invaginations (Supplementary Material, Fig. S6A). Cell death, visualized by cleaved Caspase3 immunostaining, is evident in *Six3^fl/fl^* and *Six3*^+*/fl*^*; Prop1-cre* controls in the oral ectoderm at the point where Rathke’s pouch has separated, as expected. In some mutants, the *Six3^fl/fl^; Prop1-cre* mutants had ectopic cell death extending dorsally into Rathke’s pouch (Supplementary Material, Fig. S6B). CCND1 marks proliferating cells in Rathke’s pouch as well as within the hypothalamus. CCND1 immunostaining was absent in the oral ectoderm of *Six3^fl/fl^; Prop1-cre* mutants that had poor pouch development, but it was present in mutants that had thickened oral ectoderm with multiple invaginations (Supplementary Material, Fig. S6C). At E14.5, the developing mutant pituitary gland remains highly dysmorphic and hypoplastic (Supplementary Material, Fig. S6D).

### *Six3* expression in the hypothalamus is essential for pituitary gland development

We generated hypothalamus-specific *Six3* knockout mice (*Six3^fl/fl^; Nkx2.1-cre*) to clarify the role of hypothalamic *Six3* expression in pituitary development. The efficacy and penetrance of *cre*-mediated recombination with the *Nkx2.1-cre* strain is excellent (70). At E10.5, the invagination of the oral ectoderm was similar in *Six3^fl/fl^; Nkx2.1-cre* progeny and the other genotypes, but the mutants had a more acute bend in the oral ectoderm than the other genotypes (Supplementary Material, Fig. S7A). LHX3 was not expressed (*N* = 1/3) or expressed in only a few cells in the rostral and ventral side of invaginated oral ectoderm (*N* = 2/3) (Supplementary Material, Fig. S7B). The area of PITX1 expression on the rostral side of the invaginated oral ectoderm was normal in *Six3^fl/fl^; Nkx2.1-cre* mutants, but it was weaker on the ventral side than in other genotypes.

**Figure 5:**
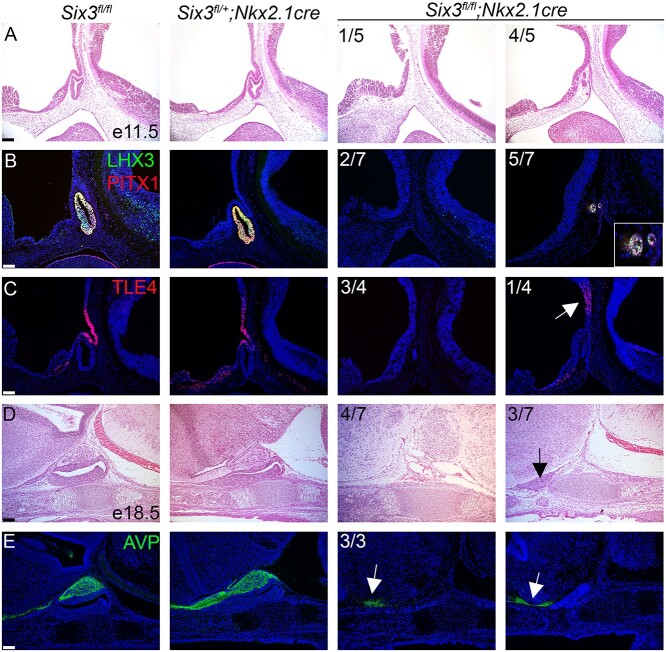
Hypothalamus-specific *Six3* knockout embryos have severe pituitary hypoplasia and stalk disruption**.** (**A**) Hematoxylin and eosin staining of sagittal sections at e11.5 reveals variable pituitary gland hypoplasia in *Six3^fl/fl^*; *Nkx2.1-cre* embryos. (**B**) LHX3 and PITX1 immunostaining are reduced in *Six3^fl/fl^*; *Nkx2.1-cre* embryos at e11.5. (**C**) TLE4 immunostaining ranged from absent or reduced on in the distal side of the infundibulum (white arrow) in *Six3^fl/fl^*; *Nkx2.1-cre* embryos. (**D**, **E**) *Six3^fl/fl^*; *Nkx2.1-cre* mice have PSIS at E18.5. (D) *Six3^fl/fl^*; *Nkx2.1-cre* mice had no pituitary or small anterior pituitary gland without a posterior lobe (black arrow). (E) All *Six3^fl/fl^*; *Nkx2.1-cre* mutants showed AVP immunopositive cells that do not project to the pituitary gland (white arrows). Two examples from the *Six3^fl/fl^*; *Nkx2.1-cre* mutants are shown for each marker, with the panel to the left representing the more severe phenotype. Incidence of observation is shown in the upper left corner of all mutant panels. Scale bars: 100 μm.

At E11.5, the *Six3^fl/fl^; Nkx2.1-cre* embryos had no obvious formation of infundibulum and no Rathke’s pouch structure, or only a tiny rudiment marked by PITX1 and LHX3 (Fig. 5A and B). TLE4 is expressed in the infundibulum and is important for mediating repression (Fig. 5C) (71). TLE4 immunostaining was either absent in *Six3^fl/fl^; Nkx2.1-cre* mutants (*N* = 3/4 samples) or was weakly expressed dorsal to the expected area in the infundibulum (*N* = 1/4 samples) (Fig. 5C).

At E18.5, a portion of the *Six3^fl/+^; Nkx2.1-cre* embryos exhibit dysmorphology (Supplementary Material, Fig. S7C). The *Six3^fl/fl^; Nkx2.1-cre* have either no anterior lobe, or a very tiny one and no posterior lobe (Fig. 5D, Supplementary Material, Fig. S7C). The remaining pituitary tissue stained with LHX3, consistent an anterior lobe identity (Supplementary Material, Fig. S7D), but there was little or no PITX1 staining (Supplementary Material, Fig. S7E). At E18.5, all *Six3^fl/fl^; Nkx2.1-cre* mutants had AVP-positive cells in the hypothalamus; however, these cells are not properly positioned in the mutants (Fig. 5E). The failure of AVP neurons to project to the posterior lobe and the poor development of the anterior lobe is consistent with PSIS. Interestingly, two out of eight of the *Six3^fl/fl^; Nkx2.1-cre* embryos had cleft palate (Supplementary Material, Fig. S7F). We analyzed the genotypes of P1 mice, but there were no *Six3^fl/fl^; Nkx2.1-cre* mutants among the 55 pups born (*P* = 0.0001; *Six3^+/fl^;*= 15, *Six3^fl/fl^* = 17, *Six3^+/^fl; Nkx2.1-cre* = 23 and *Six3^fl/fl^; Nkx2.1-cre* = 0). Thus, *Six3^fl/fl^; Nkx2.1-cre* embryos are not viable, possibly due to a lack of pituitary hormone production (72).

Since no pituitary tissue was detectable after E11.5, we analyzed proliferation and cell death to determine the mechanism(s) underlying the disappearance of the pituitary primordium. Apoptotic cells, defined as cleaved caspase-3-positive cells, were increased in the invaginated oral ectoderm, especially in the caudal side, in *Six3^fl/fl^; Nkx2.1-cre* embryo at E10.5 (Supplementary Material, Fig. S8A). At E11.5, apoptotic cells were still present in *Six3^fl/fl^; Nkx2.1-cre* embryos that had invaginated oral ectoderm and a small Rathke’s pouch, but none were detected in embryos without Rathke’s pouch (Supplementary Material, Fig. S8B). There was no evidence of infundibulum formation in *Six3^fl/fl^; Nkx2.1-cre* embryos at E11.5. The ventral diencephalon of *Six3^fl/+^, Six3^fl/fl^* and *Six3^fl/+^; Nkx2.1-cre* embryos had very few Ki-67 positive cells in the infundibulum, as expected for cells undergoing differentiation. On the other hand, *Six3^fl/fl^; Nkx2.1-cre* embryos had a homogeneous distribution of proliferating cells, suggesting that ventral diencephalon did not become patterned appropriately for cells to leave the cell cycle and differentiate into pituicytes (Supplementary Material, Fig. S8C). The expression of N-Myc (MYCN) was more robust in mutants than other genotypes in the diencephalon (Supplementary Material, Fig. S8D).

OTX2 expression in the ventral diencephalon is required for pituitary growth (70). *Six3^fl/fl^; Nkx2.1-cre* embryos did express *Otx2* in the expected area of the infundibulum and diencephalon (Supplementary Material, Fig. S8E). *Otx2^fl/fl^; Nkx2.1-cre* embryos had higher proliferation of hypothalamic cells (BrdU-positive) than other genotypes (70). To clarify the involvement of *Otx2* in *Mycn* expression in the hypothalamus, we analyzed *Otx2^fl/fl^; Nkx2.1-cre* mice. *Otx2^fl/fl^; Nkx2.1-cre* embryos had normal expression of *Mycn* at E11.5, suggesting that *Otx2* does not regulate *Mycn* in this context (Supplementary Material, Fig. S8F).

**Figure 6:**
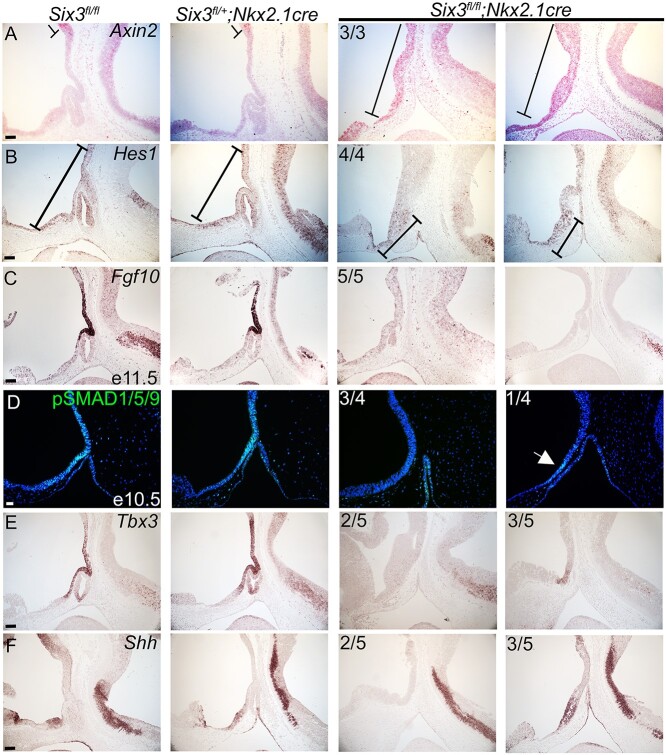
Altered patterning of the ventral diencephalon in *Six3^fl/fl^*; *Nkx2.1-cre* mice underlies pituitary hypoplasia and agenesis. (**A**) *Axin2* RNAscope detects anterior extension of *Axin2* expressing area in *Six3^fl/fl^; Nkx2.1-cre* mutants. (**B**) *Hes1* in situ hybridization detects transcripts only in the rostral part of the ventral diencephalon in *Six3^fl/fl^*; *Nkx2.1-cre* embryos*.* (**C**) *Fgf10* in situ hybridization did not detect transcripts in 5/5 *Six3^fl/fl^*; *Nkx2.1-cre* embryos. (**D**) Immunostaining for pSMAD 1/5/9, a marker of BMP signaling, was weakly positive (one out of four samples) or absent in the diencephalon of mutants. (**E**) *Tbx3* in situ hybridization is absent or reduced in *Six3^fl/fl^*; *Nkx2.1-cre* embryos. (**F**) *Shh* in situ hybridization in the diencephalon ranges from absent to comparable in *Six3^fl/fl^*; *Nkx2.1-cre* embryos. Two examples from the *Six3^fl/fl^*; *Nkx2.1-cre* mutants are shown for each marker, with the panel to the left representing the more severe phenotype. Incidence of observation is shown in the upper left corner of all mutant panels. Scale bars: 100 μm.

Retinal homeobox protein (RAX) is a transcription factor that is involved in the early patterning of the mammalian hypothalamus (73). *Rax* knockout mice form Rathke’s pouch, but infundibulum development is impaired*. Rax* expression is similar between genotypes at E11.5, suggesting that *Rax* is not dependent upon *Six3* (Supplementary Material, Fig. S9A). We hypothesized that poor infundibulum development and reduced inductive signals emanating from it caused the failure of Rathke’s pouch to develop beyond the initial invagination step. Multiple mouse studies support the roles of the WNT, NOTCH, FGF, BMP and SHH signaling from the ventral diencephalon promoting the survival and growth of pituitary progenitor cell in the Rathke’s pouch (74). RNAscope revealed that expression of *Axin2* expanded rostrally into the diencephalon in the *Six3^fl/fl^; Nkx2.1-cre* embryos, suggesting that SIX3 is required in the diencephalon to repress WNT signals (Fig. 6A). MYCN is downstream of canonical WNT signaling (75,76). Excess canonical Wnt signaling interferes with the differentiation of the ventral diencephalon into the infundibulum/posterior lobe (77,78).

Notch signaling regulates cell fate decisions in a variety of organisms and tissues. *Hes1*, a gene downstream of Notch signaling, is required for normal placement of AVP neurons (79). *Hes1* deficiency reduces LHX3 expression in the caudal side of Rathke’s pouch (80). The effect of *Hes1* knockout on cell proliferation is controversial, but there is one report of cell death of the caudal side of the invaginated oral ectoderm (80). The pattern of cell death and abnormal LHX3 expression in *Six3^fl/fl^; Nkx2.1-cre* at E10.5 is like the *Hes1* knockout embryos, prompting investigation of *Hes1* expression in our model. *Hes1* is expressed throughout the hypothalamus in control embryos; however, it is only expressed in the rostral part of the diencephalon in *Six3^fl/fl^; Nkx2.1-cre* embryos (Fig. 6B).

Both *Fgf8* and *Fgf10* expression in the ventral diencephalon are required for growth of Rathke’s pouch. *Fgf8* expression activates *Lhx3* expression in the pouch rudiment and stimulates it to grow into a mature Rathke’s pouch (81). *Fgf10* knockout mice undergo normal invagination of the oral ectoderm but lack Rathke’s pouch because of extensive apoptosis by E13.5 (82). *Six3^fl/fl^; Nkx2.1-cre* embryos had little or no detectable expression of *Fgf10* or *Fgf8* in the diencephalon (Fig. 6C, Supplementary Material, Fig. S9B). Poor expression of FGFs in the diencephalon is sufficient to explain the cell death and failure of Rathke’s pouch to develop in *Six3^fl/fl^; Nkx2.1-cre* embryos.

LIM Homeobox 2 (*Lhx2*) regulates the specification of the infundibulum and organization of differentiated cells within the anterior and intermediate lobes of the pituitary (83). *Lhx2* knockout mice had increased cell proliferation in the infundibulum with failure of the neuroectoderm to evaginate to form the posterior lobe (83). *Lhx2* is thought to act downstream of FGFs (84). *Six3^fl/fl^; Nkx2.1-cre* embryos had undetectable (*N* = 3/5) or weak expression (*N* = 2/5) of *Lhx2* in the infundibulum, implying that low *Lhx2* expression could contribute to the failure of posterior lobe formation (Supplementary Material, Fig. S9C).

Bone morphogenetic protein 4 (BMP4) is expressed in the ventral diencephalon and is another essential signaling molecule and induces the formation of Rathke’s pouch from the oral ectoderm (85). Expression of phospho-Smad1/5/9, a marker of Bmp4 signaling, was weak or not detectable in *Six3^fl/fl^; Nkx2.1-cre* embryos (Fig. 6D).

TBX3 represses the activation of *Shh* by SOX2 at the distal *Shh* enhancer, SBE2. *Tbx3*-deficient mice lack a posterior lobe, and Rathke’s pouch degenerates (84). *Six3^fl/fl^; Nkx2.1-cre* embryos showed no (*N* = 2/5) or weak (*N* = 3/5) *Tbx3* expression in the ventral diencephalon (Fig. 6E). *Tbx3* knockout mice exhibit activation of *Shh* expression in the diencephalon (67). However, *Six3^fl/fl^; Nkx2.1-cre* embryos had no (*N* = 2/5) or normal (*N* = 3/5) *Shh* expression in the diencephalon (Fig. 6F). This suggests that low expression of *Tbx3* is not the direct cause of the malformed infundibulum and high cell proliferation in the diencephalon of the *Six3^fl/fl^; Nkx2.1-cre* embryos. We examined the expression of *Six6* and found no obvious compensation in the mutants (Supplementary Material, Fig. S9D). Our data support a role for SIX3 within the oral and neural ectoderm in patterning the ventral diencephalon and promoting pituitary growth.

## Discussion

Through analysis of patient variants and mouse modeling studies, we have refined the role of SIX3 in pituitary gland development and disease. We identified a familial case of siblings with CPHD who were doubly heterozygous for variants in *SIX3* and *POU1F1* that reduce transactivation and alter splicing, respectively [this report and (67)]. Variants that alter *POU1F1* splicing to favor the repressive POU1F1-beta isoform instead of the activating POU1F1-alpha isoform cause dominant CPHD or IGHD with incomplete penetrance (49–51). While the role of POU1F1 has been well defined in pituitary disease, less is known about the role of SIX3. The SIX3 p.P74R/+ variant has weaker transactivation activity in cell culture assays than wild type but stronger activation activity than SIX3 variants that cause alobar type HP. The thin stalk observed in the affected children may be a mild form of PSIS (55), and other genes associated with HPE might also cause PSIS and CPHD. The unaffected father and one sibling with visual impairment carried the SIX3 p.P74R/+ variant, but they did not have CPHD. This could be due to incomplete penetrance of the SIX3 variant, although we cannot rule out the contribution of other genetic or environmental factors in the children with CPHD. Because the children who were doubly heterozygous for SIX3 and POU1F1 variants had CPHD, we expect that SIX3 plays a role as a second hit in causing the thin pituitary stalk phenotype.

There are many examples of digenic interactions that lead to a disease phenotype, including HPE, retinitis pigmentosa, hirschsprung disease, glaucoma and insulin resistance (37,86–91). There is also mounting evidence of digenic disease in patients with hypopituitarism and clear evidence of genetic background effects on the severity of pituitary disorders in mice (4,44,92–95). Our observations extend this paradigm to include *Six3* and *Pou1f1.* We detected digenic interactions between SIX3 and POU1F1 in mice doubly heterozygous for the loss of function alleles, *Six3*^+/−^ and *Pou1f1^+/dw^*. We discovered that SIX3 is required for the shape of the pituitary gland and palatal ossification, while SIX3 and POU1F1 together regulate pituitary gland growth and affect viability. We demonstrated that *Six3* is expressed in progenitors of the developing hypothalamus and pituitary in mice, and it co-localizes with expression of genes that are essential for differentiation of these progenitors, i.e. *Tbx3, Lhx2* and *Prop1*. We hypothesize that *Six3*^+/−^ mutations alter the PROP1-expressing progenitors such that later in development, in the context of the *Pou1f1^+/dw^* mutation, defects in progenitor cell growth are observed. This phenomenon has been described as ‘intrinsic’ buffering, where two genes play roles in the same genetic pathway at different steps, and alterations in both steps of the pathway lead to a phenotype (96,97). Alternately, SIX3 and POU1F1 might play roles in independent pathways that affect pituitary development such that alterations in each component impacts organ development.

**Figure 7:**
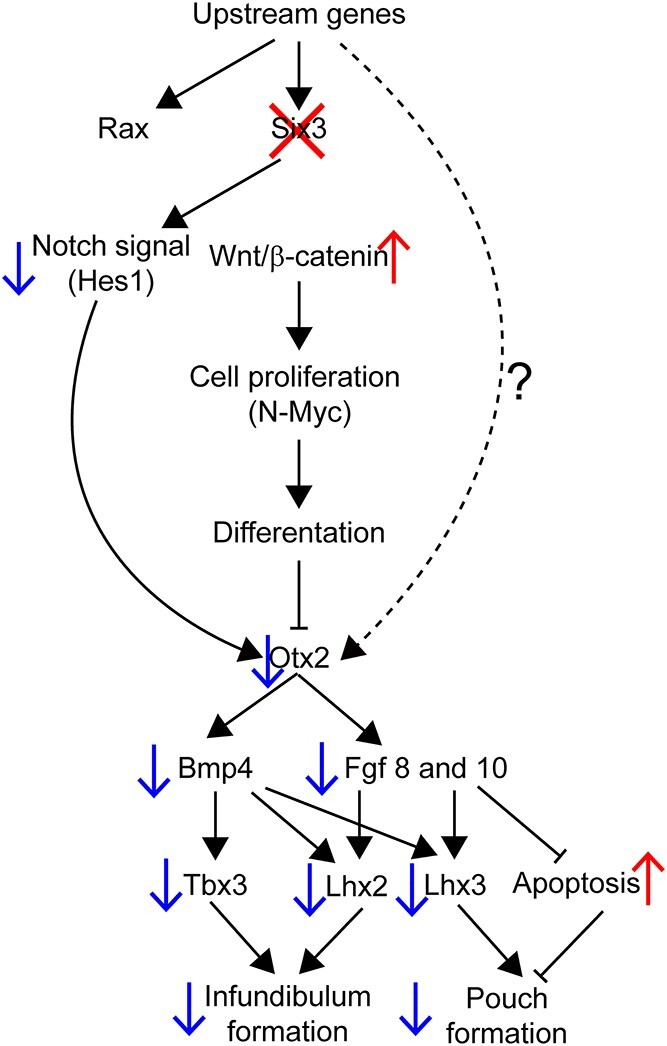
Genetic hierarchy of SIX3 action in the ventral diencephalon that affects infundibulum formation and Rathke’s pouch expansion. Deletion of *Six3* in the ventral diencephalon has no effect on *Rax* expression, but it permits elevated canonical WNT signaling and reduces Notch signaling (*Hes1*). This causes reduced expression of *Otx2,* which reduces downstream signaling by BMP and FGF, and failure to activate signature transcription factor expression for infundibulum development, namely *Tbx3* and *Lhx2.* It is also associated with reduced expression of the pituitary fate marker *Lhx3,* and apoptosis of Rathke’s pouch.

We established the position of *Six3* in the genetic hierarchy of regulating development of the ventral diencephalon into the posterior pituitary gland and hypothalamus by disrupting both alleles of *Six3* with *Nkx2.1-cre* (Fig. 7). *Six3* acts independently of *Rax*, and it is required for Notch-mediated expression of *Hes1*, suppression of proliferation (MYCN), and activation of differentiation through regulation of *Tbx3* and *Lhx2*. *Six3* is necessary for expression of *Otx2*, which is in turn necessary for stimulating BMP and FGF signaling. *Otx2^fl/fl^*; *Nkx2.1-cre* mutants had no changes in *Shh* expression (70). Thus, the reduction in *Shh* expression in *Six3* mutants must involve other *Six3* target genes. BMP, FGF and SHH signaling are necessary for activation of *Tbx3* and *Lhx2* in the infundibulum and *Lhx3* in the pouch. The *Six3^fl/fl^; Nkx2.1-cre* embryos had weak expression of *Tbx3* and *Lhx2,* but high MYCN expression in the ventral diencephalon. Several *in vitro* studies showed that overexpression of *Six3* is sufficient to downregulate MYCN (98), and *Lhx2* modulates the epigenomic profile of several cell proliferation markers, including MYCN (99). Moreover, *Six3* suppresses the Wnt/β-catenin pathway (100,101), which normally activates *Mycn* (75), and *Six3^fl/fl^; Nkx2.1-cre* embryos had high expression of MYCN and anteriorly extended *Axin2* expression. *Tbx3* knockout mice had elevated SHH signaling (43), and we observed a reduction in *Shh* activation. Thus, *Six3* is upstream of *Otx2, Hes1, Lhx2* and *Tbx3* in regulating infundibulum development.

**Figure 8:**
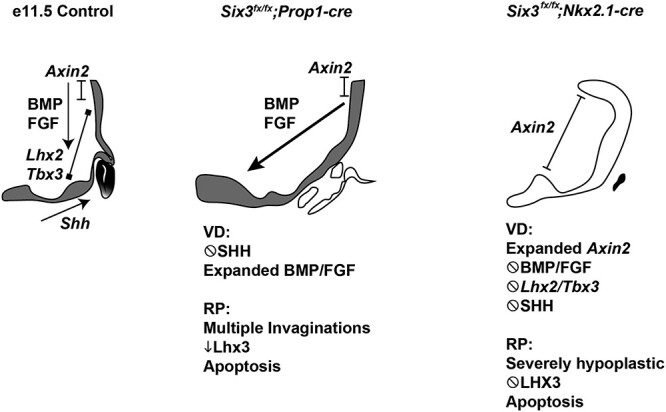
SIX3 expression is required in the oral ectoderm and neural ectoderm for the anterior and posterior lobes of the pituitary gland and the pituitary stalk. *Six3* expression is indicated at e11.5 in shaded areas. The transcription factors *Lhx2* and *Tbx3* are critical in the neural ectoderm, and *Lhx3* is essential for Rathke’s pouch development. WNT, BMP, FGF and SHH signaling stimulates growth of Rathke’s pouch and anterior pituitary development. Disruption of *Six3* expression in Rathke’s pouch with *Prop1-cre* and in the ventral diencephalon using *Nkx2.1-cre* reveals that *Six3* has critical role in both tissues*.* The mechanism of action in each tissue has similarities and differences. *Six3* is required to activate lineage specific transcription factors in each tissue, i.e. *Lhx3* in Rathke’s pouch and *Lhx2* and *Tbx3* in the ventral diencephalon. Both knockouts caused reduced BMP, FGF and SHH signaling, but failure to suppress WNT signaling (i.e. *Axin2* expression) was only detected in the ventral diencephalon knockout.

*Six3* has essential roles in both the ventral diencephalon and Rathke’s pouch. Conditional deletion of both alleles in either tissue disrupted pituitary stalk and posterior lobe formation and caused failure of Rathke’s pouch to expand (Fig. 8). Conditional deletion of *Six3* in the ventral diencephalon disrupts the pituitary organizer, resulting in reduced signaling by FGF, BMP, SHH and canonical WNT pathways. These signaling pathways are important for growth of Rathke’s pouch and expression of pituitary cell fate transcription factors, such as LHX3 (26,102,103). Conditional deletion of *Six3* in Rathke’s pouch also caused failure of LHX3 activation, and it affected the patterning of BMP, FGF and SHH signaling from ventral diencephalon indirectly. Reduction in SHH signaling blocks LHX3 activation in the pouch and arrests pouch development (104). The nature of signaling from Rathke’s pouch to the ventral diencephalon is unknown. Direct contact between the ventral diencephalon and mesenchyme is essential for induction of Rathke’s pouch from ectoderm, and mutants had abnormally localized cells that could disrupt these contacts (105).

In conclusion, *Six3* is an essential transcription factor for formation of a robust infundibulum and pituitary gland in mice. A heterozygous reduced function *SIX3* variant and a dominant *POU1F1* variant appear to act together to cause thin pituitary stalk and CPHD. In support of this idea, haploinsufficiency for both *Six3* and *Pou1f1* produces a more severe phenotype in mice. Some new candidate genes associated with HPE have been reported recently, such as *PRDM15*, *PPP1R12A* and *RAC3* (106–108). These genes might also be candidates for PSIS. As more CPHD patients are analyzed by exome sequencing and whole genome sequencing, more cases of digenic and oligogenic disease may be uncovered.

## Materials and Methods

### Patients

The GENHYPOPIT network collected anonymized information in a database declared to health authorities in accordance with local regulations at Aix-Marseille Université (AMU)—Conception Hospital (Assistance Publique—Hôpitaux de Marseille, AP-HM), and a declaration was made to the National Commission for Data Protection and Liberties (CNIL-France): 1991429 v 0. Patients or their parents signed a written informed consent to participate. The University of Michigan Institutional Review Board (UM) found the study exempt because patient DNA samples were anonymized before exome sequencing at UM. Exome sequencing was done as described (49).

A non-consanguineous family had five children, and three of them had congenital hypoglycemia and were diagnosed with TSH and GH deficiency (II-2, II-3 and II-5). II-2 had mild dysmorphic features and attended normal school. She entered puberty at 9 1/2 years and obtained a final height of 149 cm with GH and thyroid hormone replacement. An MRI in the neonatal period indicated the possibility of PSIS or thin stalk, and at 2 years of age, her MRI showed thin stalk, anterior pituitary hypoplasia and small olfactory bulb. II-3 also had mild dysmorphic features and attended normal school. His MRI detected no abnormalities other than a thin pituitary. He entered puberty at 10 years 3 months and was prescribed GnRH antagonist therapy. He is still growing with GH and thyroid hormone replacement. II-5 had neonatal GH and thyroid hormone deficiency, some dysmorphic features and delayed language acquisition. MRI was not performed. The two children without hormone deficiencies had some syndromic features. Individual II-1 had congenital nystagmus and severe visual impairment with normal development and learning, while II-4 had congenital nystagmus and autism with normal brain MRI. The father had a son from a previous marriage who had no known medical condition. The father’s height is 180 cm and the mother’s height is 165 cm.

### Cell culture, transfection and reporter assay

3T3 cells were obtained from the American Type Culture Collection. Cells were maintained in Dulbecco’s modified eagle medium (DMEM, Gibco, Grand Island, NY, USA) containing 10% fetal bovine serum (Gibco). Human SIX3 expression vectors (wild type, P74R, F87E and V92G variants) were subcloned into pcDNA3.1+/C-DYK (Invitrogen, Carlsbad, CA, USA). SHH brain enhancer 2 (SBE2) sequence was obtained from Dr Douglas J. Epstein, U. Penn (55). This sequence was subcloned into a pGL3-enhancer plasmid (Promega, Madison, WI, USA). Forkhead box protein G1 (Foxg1)-Luc plasmid was a gift from Dr Guillermo Oliver, Northwestern U. (42). pRL-TK Renilla Luciferase (pRL-TK-Rluc, Promega) was used as an internal control, and 5.0 to 7.5 × 10^4^ cells per well were seeded in a 24-well plate 24 h before transfection. Plasmids were transiently transfected into 3 T3 cells using ViaFect Transfection Reagent (Promega, Madison, WI, USA). Forty-eight hours after transfection, cells were washed with 1× phosphate-buffered saline (PBS, pH 7.4) and treated with lysis buffer for luciferase assays. Luciferase activities were measured according to the manufacturer’s recommendation (Dual-luciferase assay system; Promega). Statistical analyses were performed using JMP Statistical Database Software version 12.2.0 (SAS Institute, Cary, NC, USA). Data are presented as mean ± standard error of the mean calculated from triplicate wells. Significant differences between samples were assessed using the analysis of variance (ANOVA). *P*-values < 0.05 were considered statistically significant.

### Mice and genotyping

All procedures using mice were approved by the University of Michigan Institutional Animal Care & Use Committee. Mice were kept on a 12-h day/night cycle and had ad lib access to water and Purina 5058 chow. *Six3*^+/−^ mice and *Six3*^*fl*/+^ mice (a gift of Dr Guillermo Oliver, Northwestern University), and we received them on outbred genetic backgrounds, CD1 and NMRI, respectively. The DW/J- *Pou1f1^+/dw^*, C57BL/6 J and B6*-Tg(Nkx2.1-cre)2Sand/J*, *Nkx2.1-cre* mice (stock number 008661) (109), and C57BL/6 J.Tg- *Gt(ROSA)26Sortm9(CAG-tdTomato)Hze*/J (stock number 007909) were obtained from Jackson Laboratory, Bar Harbor, ME. The DW/J inbred genetic background has single nucleotide polymorphisms that match C57 substrains better than other inbred strains tested (110). *Six3*^+/−^ and *Pou1f1^+/dw^* mice were backcrossed individually for two generations to C57BL/6 J mice before intercrossing. The *Six3*^*fl*/+^ mice were crossed once to C57BL/6 J before intercrossing. *Tg(Prop1-cre)^432Sac^*, referred to here as *Prop1-cre,* were generated at the University of Michigan by injecting fertilized eggs from a cross of C57BL/6 J and B6D2F1 mice and then maintained on a C57BL/6 J background (66).

PCR was used to identify the *Six3* null allele with the forward oligo (5′- CTTGGGTGGAGAGGCTATTCG-3′) and reverse allele (5’-GATCCTGCAGGTACCACTCC-3′), using the following amplification parameters: 92°C 2 min, followed by 35 cycles of 92°C, 30 s; 57.5°C, 30 s; 72°C, 30 s and 1 cycle of 72°C, 10 min *Six3* null allele produces a band of 933 bp. PCR was used for the genotyping for *Six3 flox* allele with the following primer pair (forward/reverse): 5′- CGGCCCATGTACAACGCGTATT -3′/5’-CCCCTAGCCTAACCCAAACATTCG -3′, using the following amplification parameters: 95°C 2 min, followed by 35 cycles of 95°C, 30 s; 59.2°C, 30 s; 72°C, 30 s and 1 cycle of 72°C, 4 min. *Six3* wild type and flox alleles produced 405 and 487 bp, respectively. *Pou1f1^+/dw^* genotyping was previously described (111). We mated *Six3^fl/fl^* and *Six3^fl/+^; Prop1-cre* or *Six3 ^fl/+^; Nkx2.1-cre* mice for the analysis of tissue-specific knockout mice. Samples of *Otx2 ^fl^; Nkx2.1-cre* embryos were previously collected and stored (70). Samples were fixed as previously described (112). For the EdU studies, pregnant dams were injected with 50ug/g EdU in PBS. Embryos were harvested after 2 h. Proliferation was quantitated by comparing the number of proliferating cells to the total number of DAPI cells from five sections throughout the pituitary from each sample.

### Skeletal staining

Skeletal preparations were stained with alcian blue and alizarin red to visualize cartilage and bone, respectively (113). The areas of cartilage and bone staining within the secondary palate and the basioccipital bone were calculated with Image J2, Fiji. Statistical significance was determined using Student’s t-test.

### Immunostaining

Deparaffinization and hydration of paraffin-embedded samples were performed with xylene and graded alcohol and 1× PBS. Antigen retrieval was boiling paraffin-embedded sections in 10 mM citrate for 10 min. Sections were incubated with a mix of hydrogen peroxide and methanol, followed by incubation of primary antibodies and biotinylated secondary antibodies. Signals were amplified by using Tyramide Signal Amplification Kit (Biotium, Hayward, CA, USA) with or without MOM kit (Vector Laboratories, Burlingame, CA, USA). Antibody information, dilutions and detection methods are listed in Supplementary Material, Table S2. EdU-positive cells were detected using the Click-iT EdU Alexa Fluor 488 Imaging Kit, ThermoFisher cat# C10337.

### *In situ* hybridization

In situ hybridization (ISH) was performed on sections of formaldehyde-fixed paraffin-embedded tissues as previously described (70). Probe information is described in Supplementary Material, Table S3. Briefly, all DIG-labeled RNA probes were generated with transcription using RNA polymerase and labeled with DIG RNA Labeling Mix (Roche, Indianapolis, IN, USA). After pre-hybridization, probes were hybridized to tissue sections overnight. Probe binding was detected with anti-Digoxigenin-AP, Fab fragments (Roche), and nitro-blue tetrazolium chloride/5-bromo-4-chloro-3-indolyl phosphate (NBT/BCIP, Roche).

RNAscope was performed using the protocol and reagents, including the RNAscope 2.5 HD Red Detection kit, from Advanced Cell Diagnostics (Cat#322360). Sections were treated with the hydrogen peroxide reagent for 10 min at room temperature and then rinsed in water. Antigen retrieval was performed with the target retrieval reagent in the microwave for 15 min, immediately removed and then washed in 100% ethanol. Sections were dried at room temperature. Samples were treated with Protease Plus for 30 min at 40°C, washed in MilliQ water and then incubated with probe or a negative control for 2 h. Samples were washed in 1× wash buffer, and then steps AMP1–AMP4 were performed at 40°C as per the manufacturer’s protocol washing with 1× wash buffer in between steps. Sections were incubated in AMP5 at room temperature for 30 min to 1 h depending on the probe, see notes below. After washing, sections were incubated in AMP6 for 15 min, followed by a 10 min incubation in Fast Red A:Fast Red B (60:1). After washing in MilliQ water, samples were counterstained with 1:8 dilution of hematoxylin in water. The probes used and incubation times in AMP5 are as follows: *Axin2*, cat#400331; 45 min, *Ctgf*, cat#314541, 45 min; *Cyr61*, cat#429008; *Bmp4*, cat#425011, 1 h.

### Image analysis

Images of immunostaining and ISH obtained with a DFC7000 T (Leica Microsystems, Wetzlar, Germany). Images of the skeletal preps were obtained with DFC310 FX (Leica). Images were processed using LasX software (Leica Microsystems).

### Statistical analysis

Statistical analyses were performed using JMP Statistical Database Software version 12.2.0 (SAS Institute). ANOVA, Fisher’s exact test and Pearson’s chi-square test were used as appropriate. A *P*-value of <0.05 was considered statistically significant.

### Study approval

Experiments in mice were conducted under the IACUC approval number PRO00008702. Studies on patient samples were conducted with written informed consent prior to the start of experiments. Patient studies are approved by the National Commission for Data Protection and Liberties (CNIL-FRANCE), number 1991429.

## Supplementary Material

Bando_SupplementalMaterial_revised_ddac192
