# Supplementary material for: Heterozygous variants in SIX3 and POU1F1 cause pituitary hormone deficiency in mouse and man
Source: Hum Mol Genet. 2022 Aug 11;32(3):367–85. doi: 10.1093/hmg/ddac192 (PMC9851746; doi:10.1093/hmg/ddac192)
Supplement: Bando_SupplementalMaterial_revised_ddac192 [file bando_supplementalmaterial_revised_ddac192.pdf]

Supplementary Figure 1

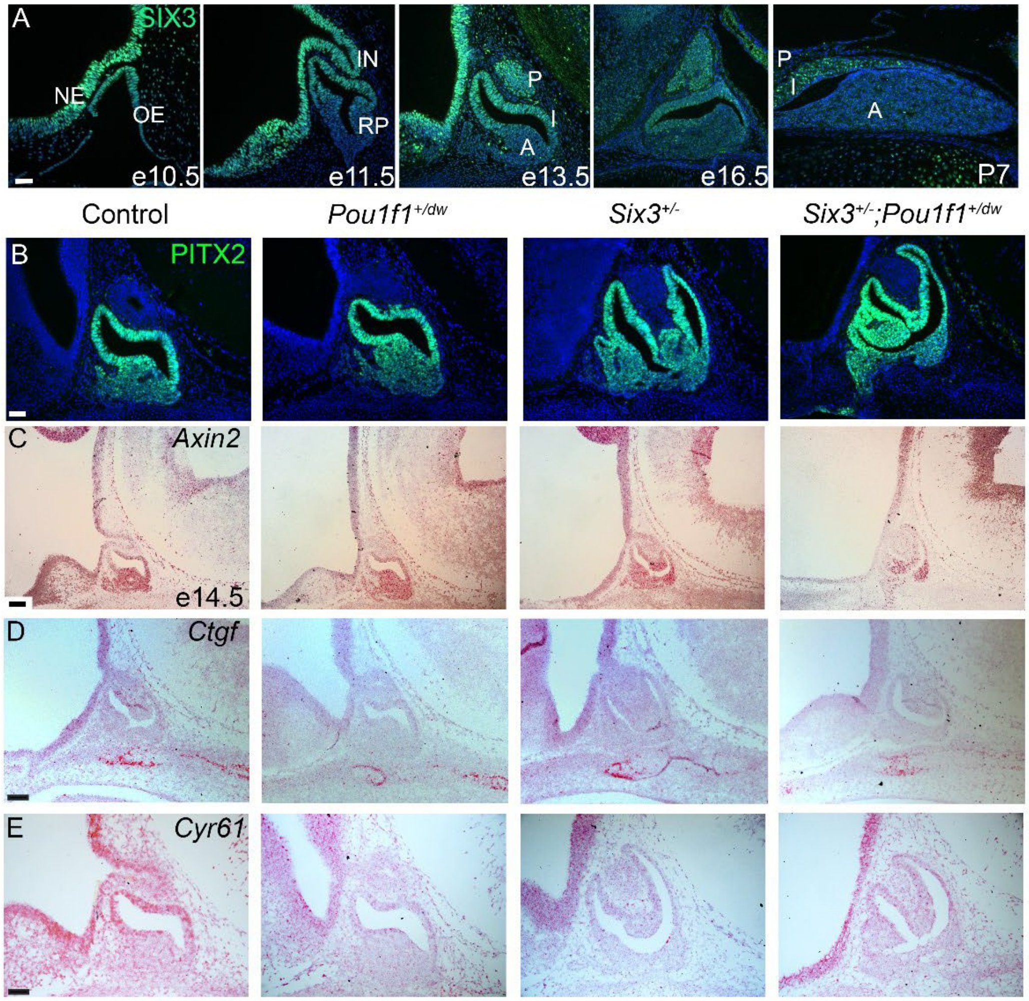

Supplemental Figure 1. Haploinsufficiency for SIX3 and POU1F1 permits pituitary cell fate acquisition. (A) SIX3 is expressed in both the hypothalamus derived from neural ectoderm (NE) and the invaginating Rathke's pouch derived from oral ectoderm (OE) at e10.5. SIX3 expression is maintained in the hypothalamus and infundibulum (IN) as well as the dorsal aspect of Rathke's pouch at e11.5. SIX3 protein can be identified in the hypothalamus as well as the forming posterior (P) and intermediate (I) lobes of the pituitary, but it is decreased in the anterior (A) lobe at 13.5. SIX3 expression is decreased at e16.5. At P7, SIX3 is predominately expressed in the intermediate lobe, with scattered cells in the anterior lobe. (B) PITX2 immunostaining, (C) *Axin2* RNA-scope in situ hybridization, (D) *Ctgf* and (E) *Cyr61* in situ hybridization do not detect differences in expression among the genotypes.

Supplemental Figure 2

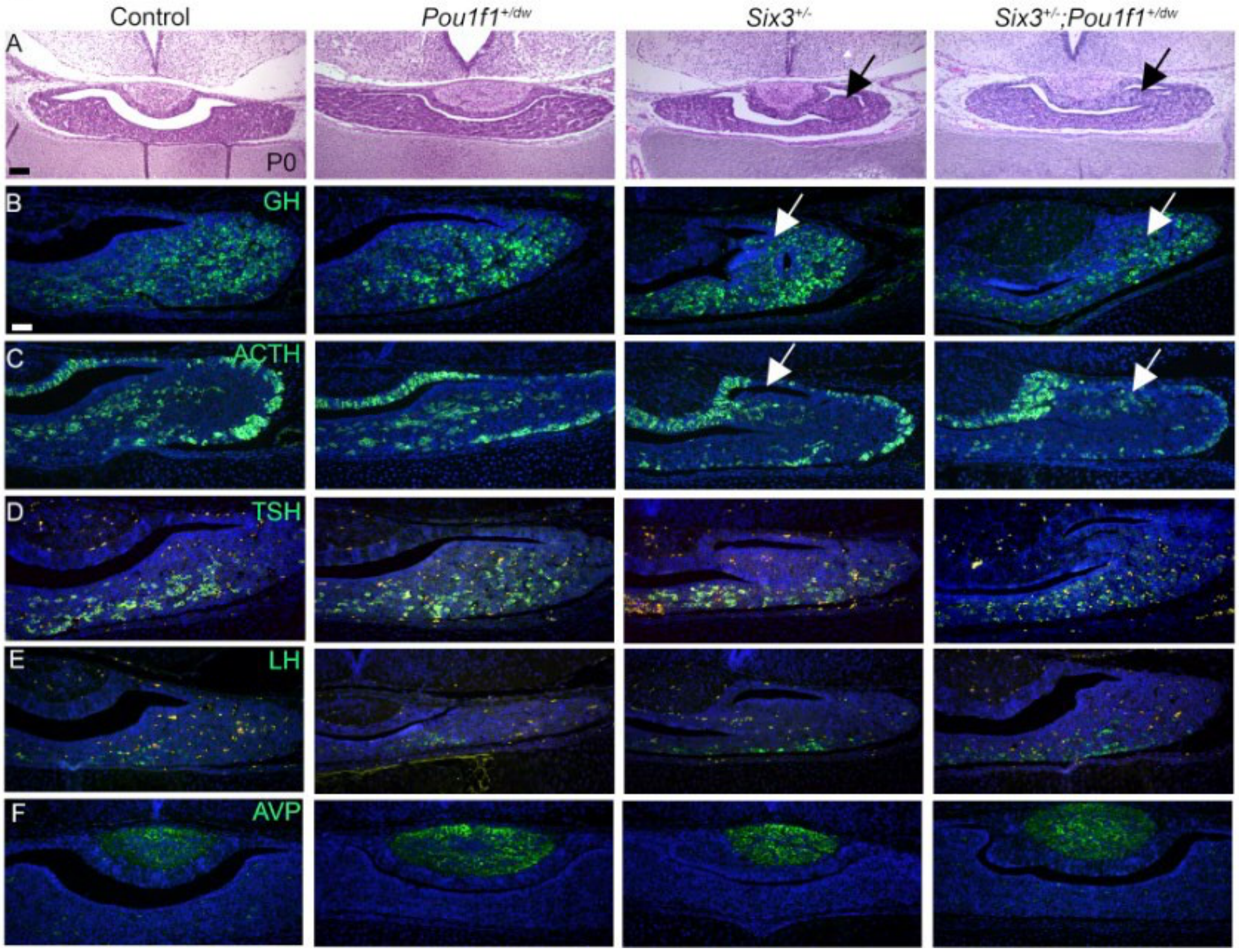

Supplemental Figure 2. Cell specification occurs in *Six3*$^{+/-}$ and *Six3*$^{+/-}$;*Pou1f1*$^{+/dw}$ despite changes in pituitary morphology.

(A) Hematoxylin and eosin staining of coronal sections from P0 mice reveals persistent dysmorphology around the marginal zone in *Six3*$^{+/-}$ and *Six3*$^{+/-}$; *Pou1f1*$^{+/dw}$ mutants. Arrows show the abnormal connection between the intermediate and anterior lobes. (B-E) Immunostaining for pituitary hormones (B) GH, (C) ACTH, (D) TSH, and (E) LH in coronal sections from newborn mice reveal GH and ACTH positive cells within the dysmorphic region of *Six3*$^{+/-}$ and *Six3*$^{+/-}$; *Pou1f1*$^{+/dw}$ mutants (white arrows). (F) Specification and projection of neurons into the posterior lobe was confirmed with AVP staining immunostaining in coronal sections of newborn mice.

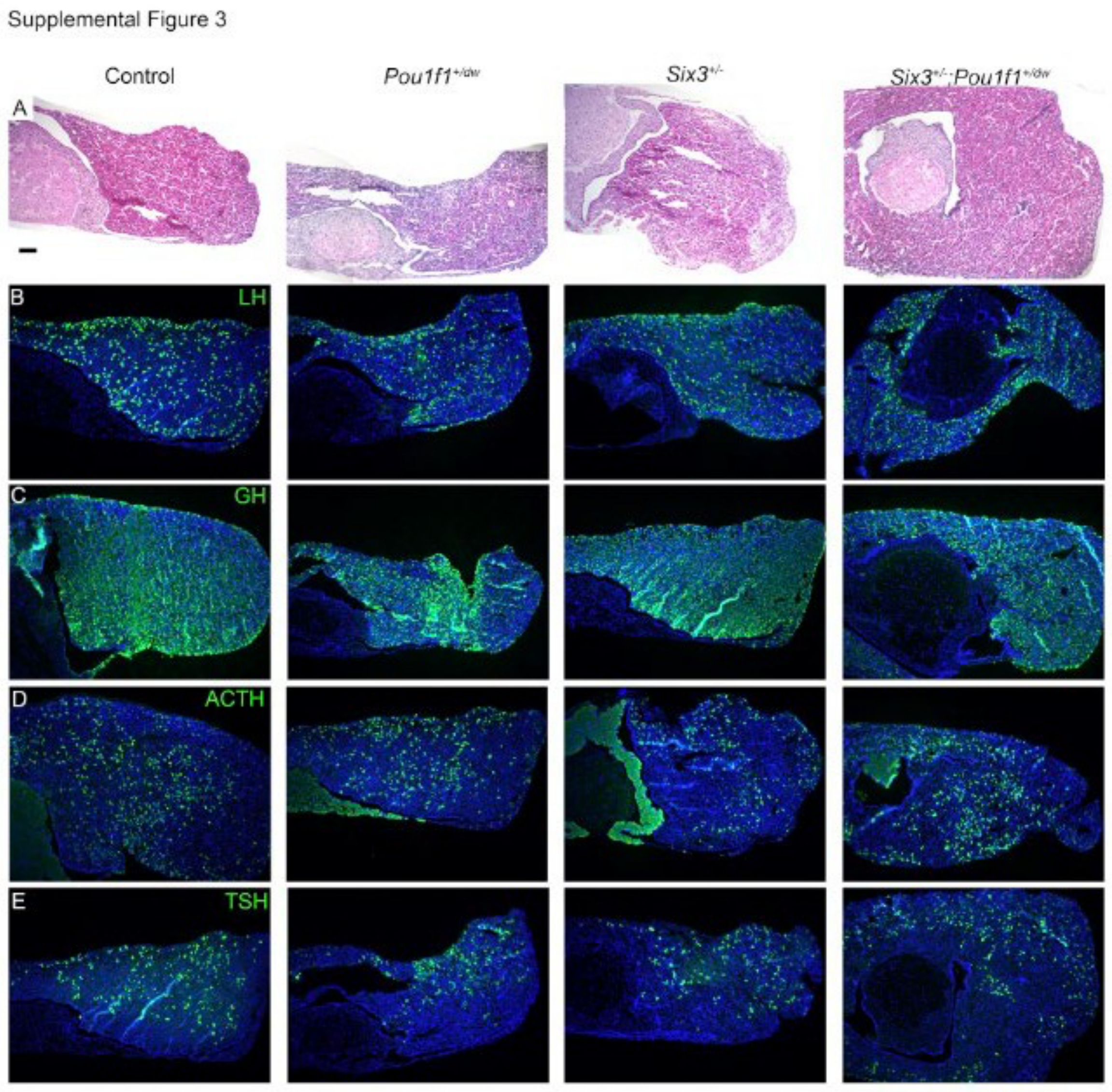

<u>Supplemental Figure 3. Cell specification in mature pituitary glands.</u>
(A) Coronal sections of pituitaries from 6-week-old mice of the indicated genotypes were stained with hematoxylin and eosin. (B-E) Pituitary hormone expression in adult mice was detected by immunostaining with hormone-specific antibodies for (B) LH, (C) GH, (D) POMC, and (E) TSH. The scale bar in panel A represents 100 μm and is applicable to all panels.

Supplemental Figure 4

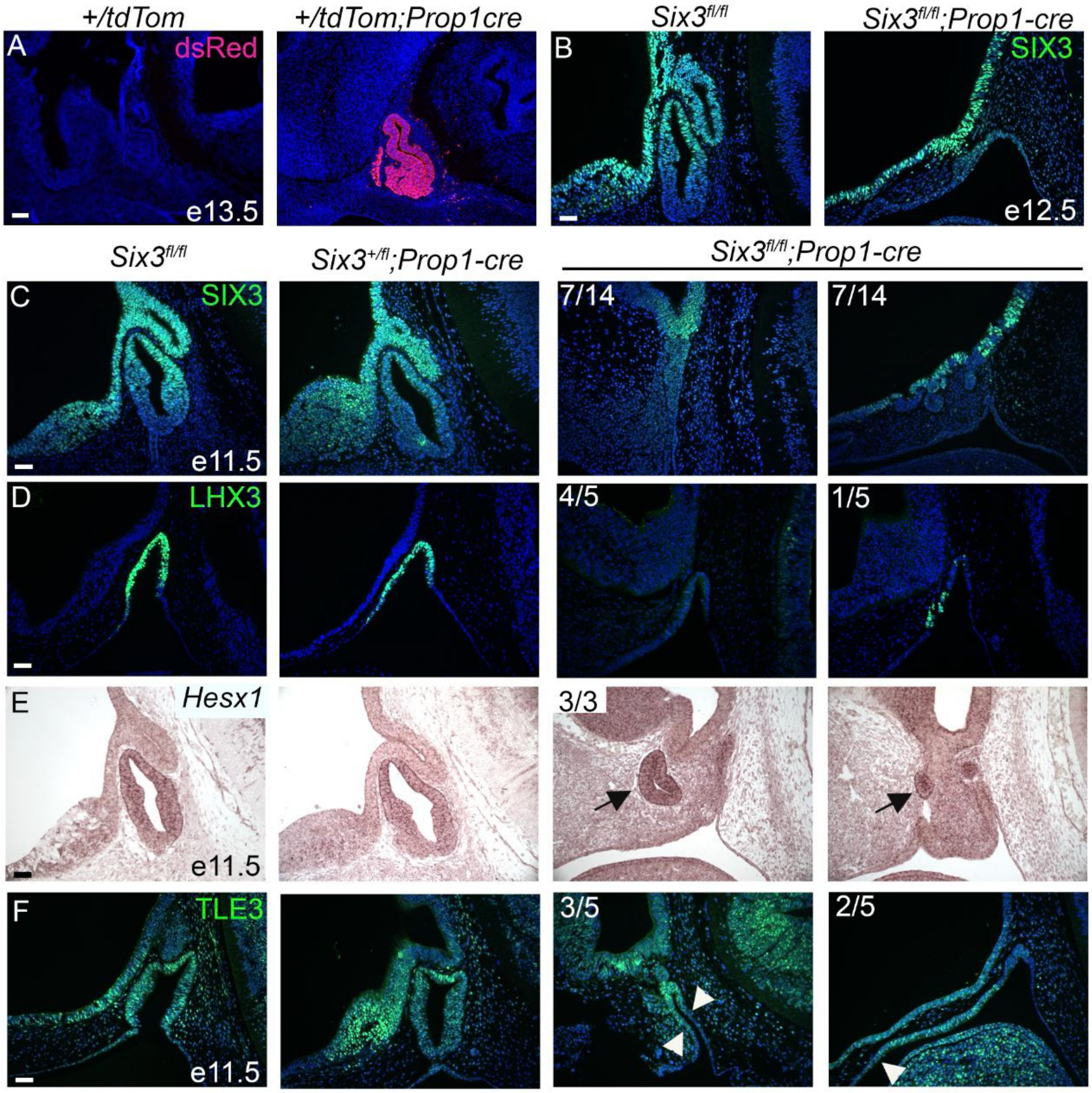

<u>Supplemental Figure 4. Validation of *Prop1-cre* specificity and analysis of gene expression in pituitary-specific knockout of *Six3.*</u>

(A) dsRed antibody staining of *+/tdTomato; Prop1-cre* e13.5 embryos reveals specific reporter expression in Rathke's pouch. SIX3 immunostaining in sagittal sections collected at e12.5 (B) and e11.5 (C) shows loss of SIX3 in Rathke's pouch of $Six3^{fl/fl}$; *Prop1-cre* embryos and retention of expression in the ventral diencephalon. (D) LHX3 immunostaining at e10.5 shows lack of expression in most mutants. (E) *Hesx1* expression was detected by in situ hybridization at e11.5, and arrows indicate transcripts detected in the mutants. (F) TLE3 is detected by immunostaining at e11.5, and arrowheads indicate the boundaries of expression in the oral ectoderm of mutants. Two examples from the $Six3^{fl/fl}$; *Prop1-cre* mutants are shown for each marker, with the panel to the left representing the more severe phenotype. Incidence of observation is shown in the upper left corner of all mutant panels. The scale bar in panel A represents 100 μm. The scale bars in B-F represent 50 μm.

Supplemental Figure 5

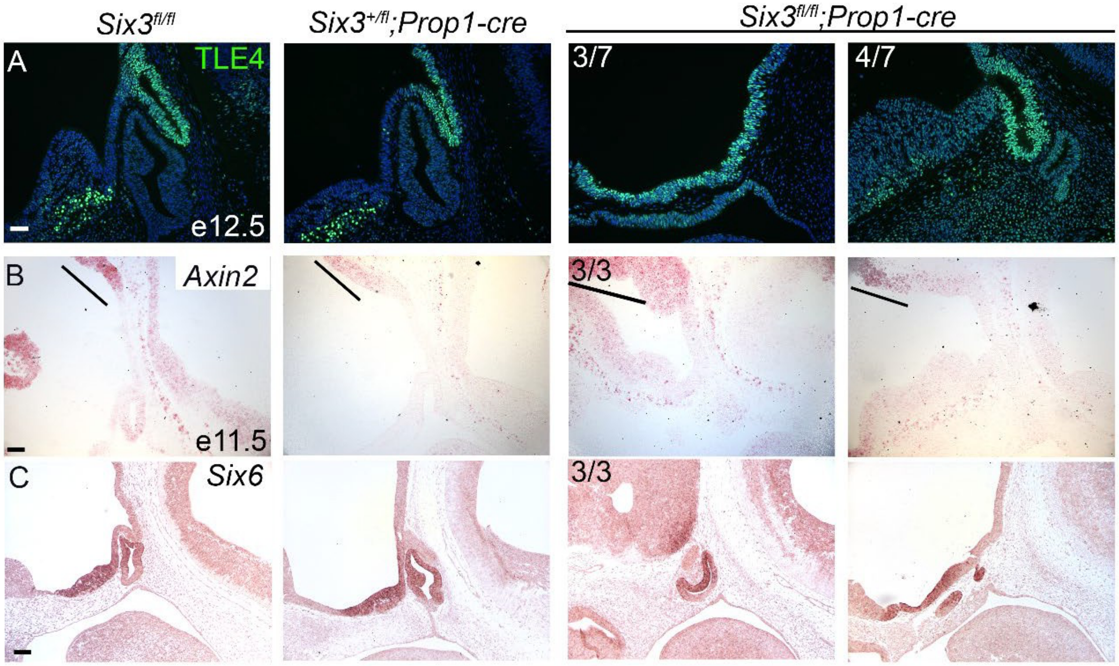

Supplemental Figure 5. *Six3*$^{fl/fl}$*; Prop1-cre* mutants have variable expression of some ventral diencephalon markers.

(A) TLE4 immunostaining is normal in mutants with a prominent infundibulum but expanded in mutants with no obvious evagination. (B) *Axin2* transcripts were detected by RNA-scope in situ hybridization. No obvious changes were noted in mutants; the black line marks the region of expression region of expression. (C) *Six6* transcripts are comparable between controls and mutant. Two examples from the *Six3*$^{fl/fl}$*; Prop1-cre* mutants are shown for each marker, with the panel to the left representing the more severe phenotype. Incidence of observation is shown in the upper left corner of all mutant panels.

Supplemental Figure 6

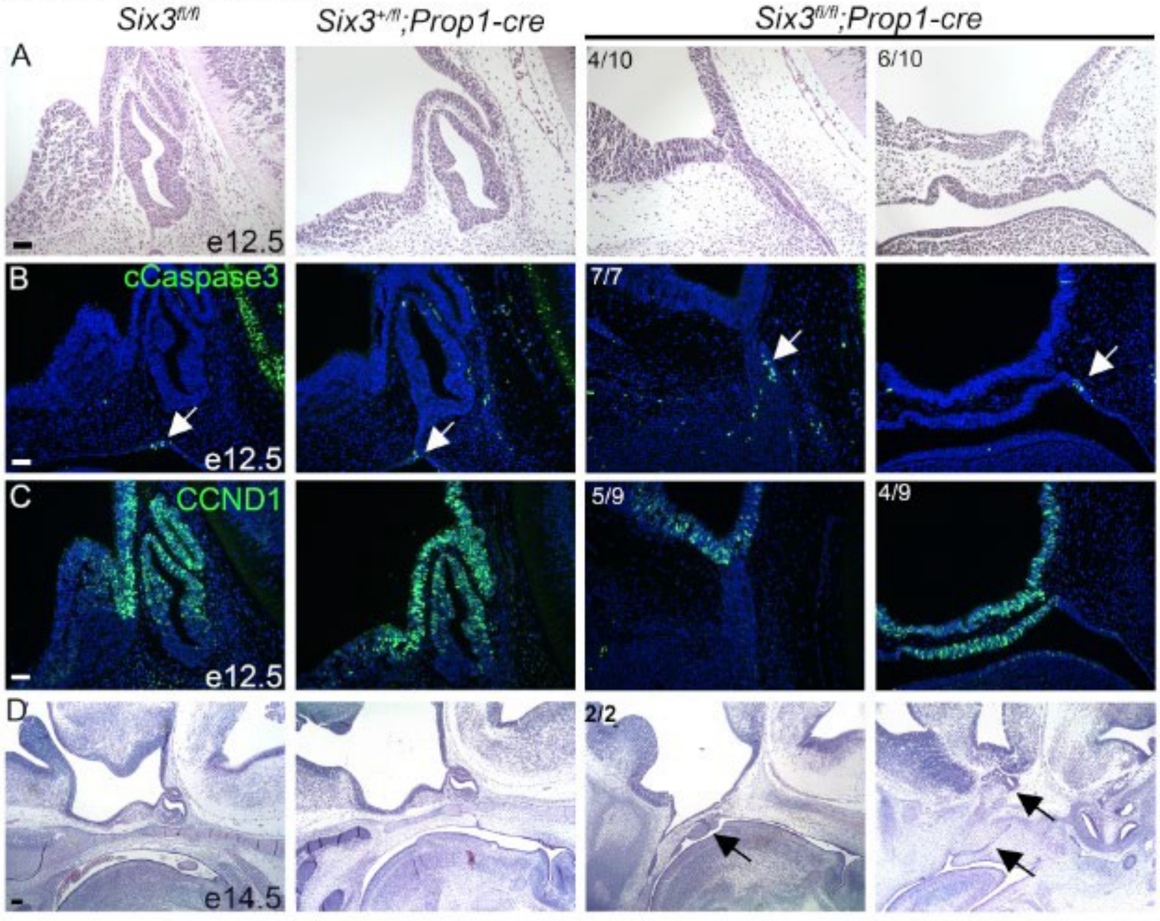

Supplemental Figure 6. The pituitary gland fails to develop in *Six3*$^{fl/fl}$*; Prop1-cre* mutants.
(A) Sagittal sections from embryos collected at e12.5 were stained with hematoxylin and eosin. The *Six3*$^{fl/fl}$*; Prop1-cre* mutants had variable levels of dysmorphology ranging from a thin hypoplastic Rathke's pouch to multiple invaginations. (B) Apoptotic cells were detected by immunostaining for cleaved Caspase3. *Six3*$^{fl/fl}$*; Prop1-cre* mutants exhibited cell death within Rathke's pouch. Arrows mark apoptotic cells. (C) CCND1 immunostaining revealed variable expression in *Six3*$^{fl/fl}$*; Prop1-cre* mutants including a failure to maintain expression in Rathke's pouch in some mutants. (D) Sagittal sections embryos collected at e14.5 were stained with hematoxylin and eosin. Rathke's pouch fails to form in the *Six3*$^{fl/fl}$*; Prop1-cre* mutants, and arrows highlight regions of possible invaginations of the oral ectoderm. Two examples of the *Six3*$^{fl/fl}$*; Prop1-cre* mutants are shown for each experiment, with the panel to the left representing the more severe phenotype. Incidence of observation is shown in the upper left corner of all mutant panels. Scale bars in panels A-C represent 50 μm. The scale bar in panel D represents 100 μm

Supplemental Figure 7

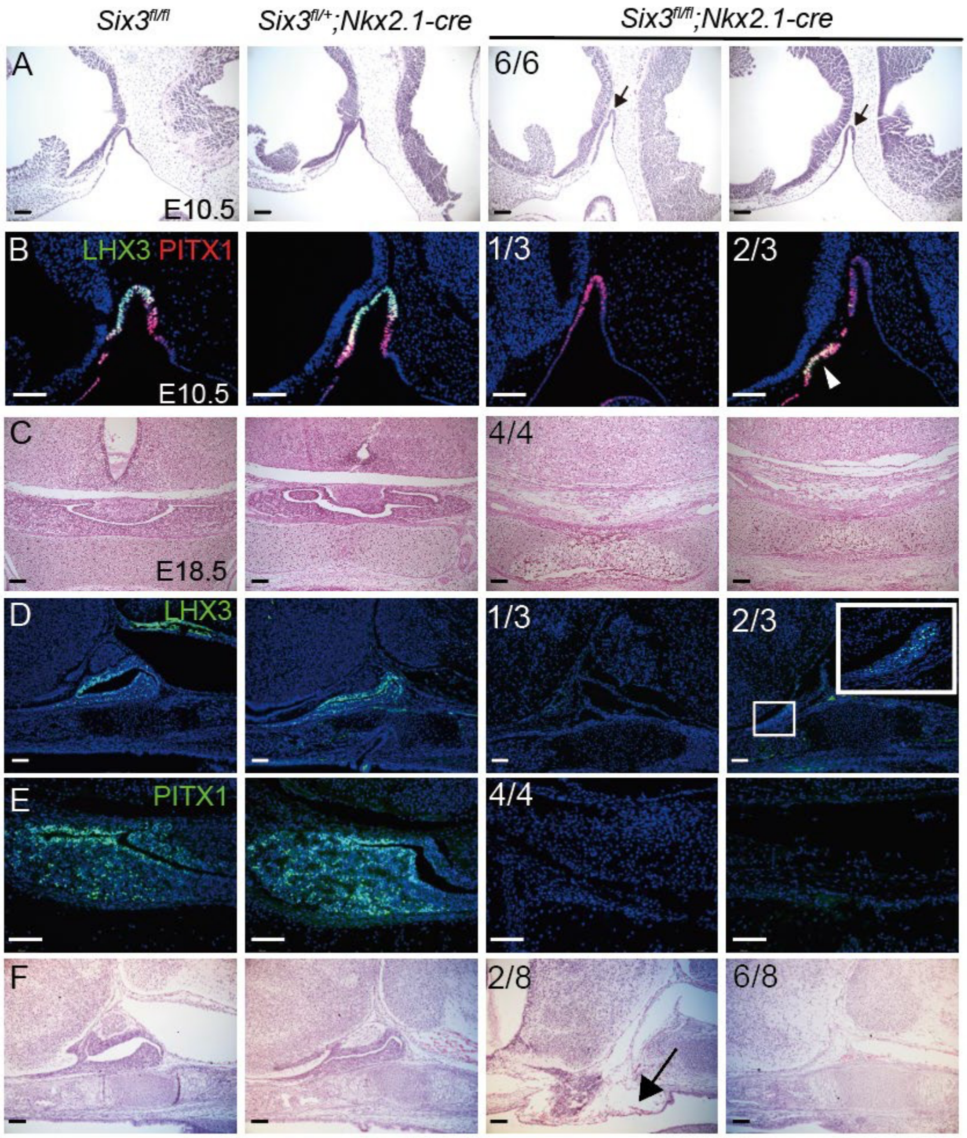

Supplemental Figure 7. Pituitary hypoplasia persists throughout embryonic development in *Six3*$^{fl/fl}$*; Nkx2.1-cre* mutants.

(A) Hematoxylin and eosin staining of sagittal sections from E10.5 embryos showed invaginated oral ectoderm in all genotypes. The invaginated oral ectoderm of *Six3*$^{fl/fl}$*; Nkx2.1-cre* mice had a more acute shape (arrows). (B) LHX3 (green) and PITX1 (red) double immunostaining at e10.5 marks Rathke's pouch. *Six3*$^{fl/fl}$*; Nkx2.1-cre* mutants range from a loss of LHX3 to reduced expression of LHX3, arrowhead. (C) Coronal sections stained with H&E at e18.5 shows loss of pituitary in *Six3*$^{fl/fl}$*; Nkx2.1-cre* mutants. (D) LHX3 immunostaining is detected in some pituitary cells of *Six3*$^{fl/fl}$*; Nkx2.1-cre* mutants collected e18.5. (E) PITX1 protein is not identified in *Six3*$^{fl/fl}$*; Nkx2.1-cre* mutants at e18.5, coronal sections. (F) H&E staining of sagittal sections at e18.5 highlight an example of cleft palate, arrow, in *Six3*$^{fl/fl}$*; Nkx2.1-cre* mutants.

Supplemental Figure 8

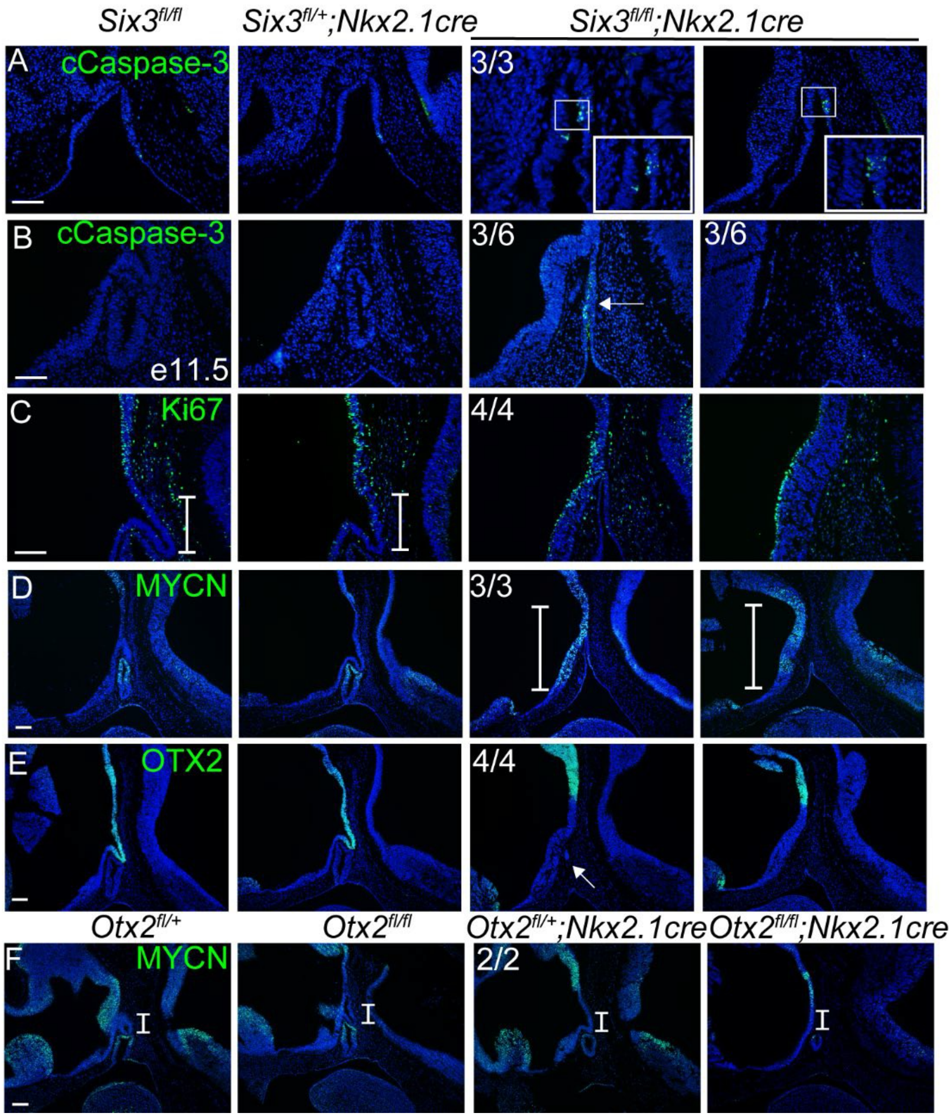

<u>Supplemental Figure 8.   Regions of proliferation are altered in hypothalamic *Six3* deletion mice.</u> **(**A) At E11.5 invaginated oral ectoderm of *Six3*$^{fl/fl}$*; Nkx2.1-cre* embryos had increased staining for cleaved Caspase3, if Rathke's pouch tissue was present. (B) Normally cells within the ventral diencephalon leave the cell cycle as they evaginate to form the infundibulum, but Ki67 positive proliferating cells were identified throughout the ventral diencephalon and infundibular area of *Six3*$^{fl/fl}$*; Nkx2.1-cre* mutants. Similar results were seen with (C) CCND1 and (D) MYCN immunostaining. Brackets denote regions in the controls that are devoid of proliferating cells. (E) OTX2 expression was shifted dorsally into the hypothalamus of *Six3*$^{fl/fl}$*; Nkx2.1-cre* mutants compared to controls. Arrow highlights the hypoplastic pituitary. (F) MYCN expression is restricted from the developing infundibulum in *Otx2*$^{fl/fl}$*; Nkx2.1-cre* mutants and controls, bracket denotes region without expression. Two examples from the *Six3*$^{fl/fl}$*; Nkx2.1-cre* mutants are shown for each marker, with the panel to the left representing the more severe phenotype. Incidence of observation is shown in the upper left corner of all mutant panels. Scale bars represent 100 μm.

Supplemental Figure 9

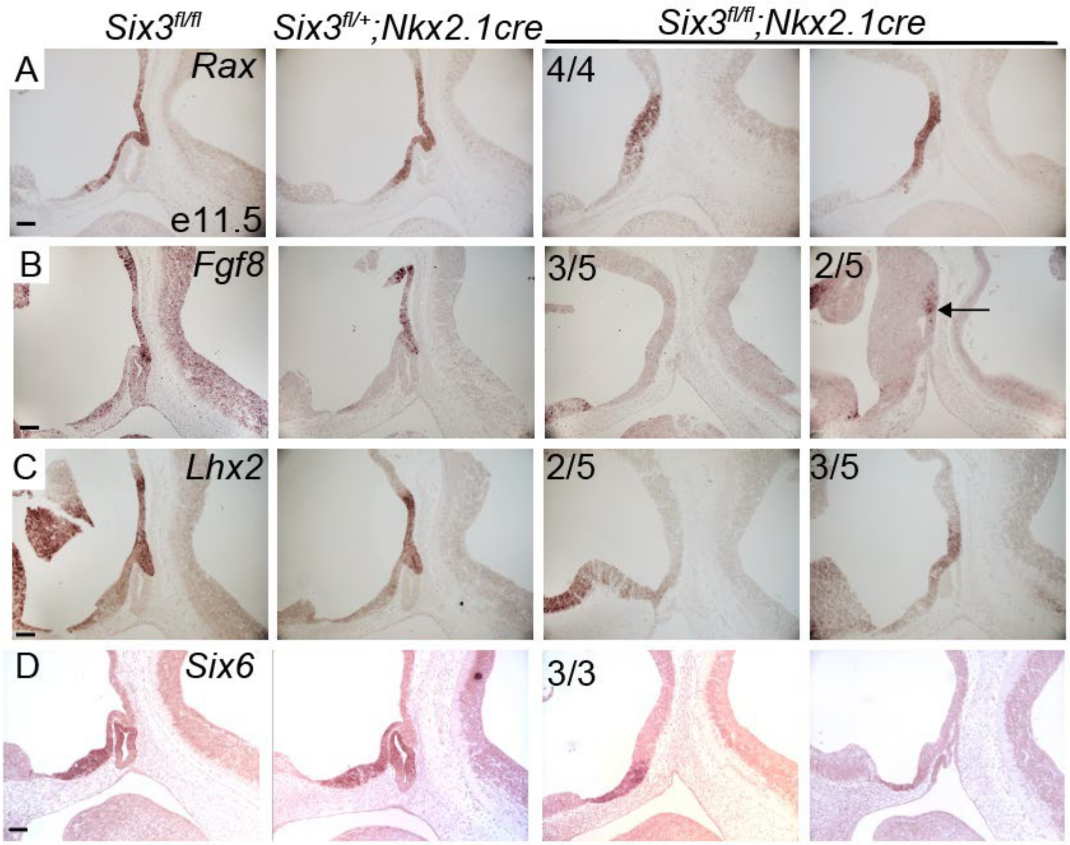

Supplemental Figure 9. In situ hybridization detects reduced expression of *Fgf8* and *Lhx2* in the infundibulum of *Six3*$^{fl/fl}$*; Nkx2.1-cre* mutants.

(A) *Rax* expression is similar between genotypes at e11.5. (B) *Fgf8* ISH. Three out of five *Six3*$^{fl/fl}$*; Nkx2.1-cre* mice had no expression of *Fgf8*, and the remaining had only a small *Fgf8*-positive region. (C) *Lhx2* ISH. Two out of five *Six3*$^{fl/fl}$*; Nkx2.1-cre* mutants had no expression of *Lhx2* in the infundibulum, and three had a small *Lhx2*-positive region. (D) *Six6* transcripts were not elevated in *Six3*$^{fl/fl}$*;Nkx2.1-cre* mutants suggesting no compensation.

Supplemental Table 1: Comparable proliferation rates in Rathke's pouch at e13.5				
Genotype	EdU positive cells	DAPI cells	Proliferation (%)	p value [1]
Control	533	3227	17	
	670	3825	17	
	750	3576	21	
Pou1f1$^{+/dw}$	666	2899	23	
	616	3031	20	
	727	3511	21	
	674	3611	19	0.21
Six3$^{+/-}$	641	2955	22	
	623	3265	19	
	994	3339	30	
	964	4452	22	
	835	3359	25	
	751	4398	17	0.11
Six3$^{+/-}$; *Pou1f1*$^{+/dw}$	800	2879	28	
	965	4260	23	
	789	3983	20	
	878	4343	20	
	861	4535	19	0.13

[1] calculated relative to control with Student's T-test, two-tailed distribution, unequal variance

Supplemental Table 2. Antibodies and Immunostaining Conditions				
Target protein	Antigen Retrieval	Blocking	Primary antibody	Secondary antibody, detection
AVP	boil 10 min citric acid	TSA block	1:500, Abcam, ab213708	Anti-rabbit Biotin (1:200, BA-1000, Vector Laboratories, #BA-1000), Biotium TSA kit
BrdU	boil 10 min citric acid	M.O.M block (Vector Laboratories)	1:100 Sigma B2531	Anti-mouse Biotin in M.O.M kit as manufacture's instruction, Biotium TSA kit
CCND1	boil 10 min citric acid	M.O.M block (Vector Laboratories)	1:200, Santa Cruz, sc-8396	Anti-mouse Biotin in M.O.M kit as manufacture's instruction, Biotium TSA kit
CCND2	boil 10 min citric acid	NGD block	1:200, Santa Cruz, sc-593	Anti-rabbit Biotin, Biotin-TSA Kit
cleaved caspase-3	boil 10 min citric acid	TSA block	1:50, Cell Signaling Technology, #9661	Anti-rabbit Biotin (1:200, Vector Laboratories, #BA-1000), Biotium TSA kit
GH	None	NGD block	1:600, National Hormone and Peptide Program	Anti-human biotin, Strep-Cy2
Ki-67	boil 10 min citric acid	TSA block	1:500, Abcam, ab15580	Anti-rabbit Biotin, Biotium TSA kit
LH	None	NGD block	1:1000, National Hormone and Peptide Program	Anti-guinea pig Biotin, Strep-Cy2
LHX3	boil 10 min citric acid	M.O.M block	1:100, Developmental Studies Hybridoma Bank, #67.4E12	Anti-mouse Biotin in M.O.M kit, Biotium TSA kit
MYCN	boil 10 min citric acid	TSA block	1:250, Cell Signaling Technology, #51705	Anti-rabbit Biotin, Biotium TSA kit
OTX2	boil 10 min citric acid	TSA block	1:500, Abcam, ab21990	Anti-rabbit Biotin, Biotium TSA kit
p57	boil 10 min citric acid	TSA block	1:100, ThermoFisher MA5-11309	Anti-mouse Biotin, Biotium TSA Kit
Phospho-Smad1 (Ser463/465)/ Smad5 (Ser463/465)/ Smad9 (Ser465/467)	Histo VT one, 90 C 40 min	TSA block	1:200, Cell Signaling Technology, #13820	Anti-rabbit Biotin, Biotium TSA kit
PITX1	10 min	TSA block	1:250, Dr. Jacques Drouin (Université de Montréal)	Anti-rabbit Biotin, Biotium TSA kit
PITX2	10 min	TSA block	1:250, Dr. Jacques Drouin (Université de Montréal)	Anti-rabbit Biotin, Biotin-TSA Kit

Supplemental Table 2 (continued). Antibodies and Immunostaining Conditions				
Target protein	Antigen Retrieval	Blocking	Primary antibody	Secondary antibody, detection
POMC	None	NGD block	1:1000, National Hormone and Peptide Program	Anti-rabbit Biotin, Strep-Cy2
POU1F1	boil 10 min citric acid	NGD block	1:100, Dr. Simon Rhodes	Anti-rabbit Alexa 555, (1:200, Life Technologies), NGD
PROP1	boil 10 min citric acid	NGD block	1:100, Dr. Aimee Ryan	Anti-guinea pig Biotin, Biotium TSA kit
SIX3	boil 10 min citric acid	TSA block	1:250, Rockland antibody, #600-401-A26S	Anti-rabbit Biotin, Biotium TSA kit
TCF4 (TCF7L2)	boil 10 min citric acid	M.O.M block	1:50, Cell Signaling Technology, #2953	Anti-mouse Biotin in M.O.M kit, Biotium TSA kit
TLE3	boil 10 min citric acid	M.O.M. block	1:100, Santa Cruz, sc-514798	Anti-mouse Biotin, Biotium TSA Kit
TLE4	boil 10 min citric acid	0.5% Triton-X/PBS + 1%BSA + 5% normal goat serum	1:1000, Dr. Stefano Stefani (McGill University)	Fluorescein (FITC) AffiniPure Donkey Anti-Rabbit IgG (H+L) (1:200, Jackson Immuno Research, 711-095-152)
TSH	None	NGD block	1:1000, National Hormone and Peptide Program	Anti-rabbit Biotin, Strep-Cy2

Supplemental Table 3. In situ hybridization probes				
Target mRNA	RNA polymerase	Restriction enzyme for Anti-sense	Hybridization temperature	Source
Fgf10	T3	BamH1	55 C	Brigid Hogan
Fgf8	T7	BamH1	59 C	Gail Martin
Hes1	T3	HindIII	55 C	Ryoichiro Kageyama
Hesx1	T3	BamH1	55 C	Paul Thomas
Lhx2	T3	Sal1	55 C	Sally Camper
Rax	T3	PCR product template	55 C	Sally Camper
Shh	Sp6	SacII	55 C	Carles Gaston-Massuet
Six6	T3	BamH1	58 C	Sally Camper
Tbx3	T3	Sal1	55 C	Sally Camper
